# Expansion of an Unusual Virtual Memory CD8^+^ Subpopulation Bearing Vα3.2 TCR in Themis-Deficient Mice

**DOI:** 10.3389/fimmu.2021.644483

**Published:** 2021-04-07

**Authors:** Mukul Prasad, Lukasz Wojciech, Joanna Brzostek, Jianfang Hu, Yen Leong Chua, Desmond Wai Hon Tung, Jiawei Yap, Vasily Rybakin, Nicholas R. J. Gascoigne

**Affiliations:** ^1^ Immunology Translational Research Programme, Yong Loo Lin School of Medicine, National University of Singapore, Singapore, Singapore; ^2^ Immunology Programme, Life Sciences Institute, National University of Singapore, Singapore, Singapore; ^3^ Department of Microbiology and Immunology, Yong Loo Lin School of Medicine, National University of Singapore, Singapore, Singapore; ^4^ Department of Immunology and Microbiology, The Scripps Research Institute, La Jolla, CA, United States

**Keywords:** bystander activation, CD8 T cell, self-reactive, themis, T cell receptor

## Abstract

Deletion of the gene for Themis affects T cell selection in the thymus, which would be expected to affect the TCR repertoire. We found an increased proportion of cells expressing Vα3.2 (TRAV9N-3) in the peripheral CD8^+^ T cell population in mice with germline *Themis* deficiency. Analysis of the TCRα repertoire indicated it was generally reduced in diversity in the absence of Themis, whereas the diversity of sequences using the TRAV9N-3 V-region element was increased. In wild type mice, Vα3.2^+^ cells showed higher CD5, CD6 and CD44 expression than non-Vα3-expressing cells, and this was more marked in cells from Themis-deficient mice. This suggested a virtual memory phenotype, as well as a stronger response to self-pMHC. The Vα3.2^+^ cells responded more strongly to IL-15, as well as showing bystander effector capability in a *Listeria* infection. Thus, the unusually large population of Vα3.2^+^ CD8^+^ T cells found in the periphery of Themis-deficient mice reflects not only altered thymic selection, but also allowed identification of a subset of bystander-competent cells that are also present in wild-type mice.

## Introduction

The inability to predict the foreign antigens that an organism will encounter in its lifetime creates a need for the immune system to generate and maintain a diverse T cell receptor repertoire (1–3). T cells develop in the thymus, where they rearrange T cell receptor (TCR) α and β genes and undergo positive and negative selection to ensure that only cells expressing TCRs which give an optimum response to the self-peptide MHC (pMHC) are allowed to leave the thymus. The TCR is formed by rearrangement of V(D)J elements, such that the binding site for the peptide MHC complex is formed from 3 complementarity determining regions (CDR1, 2, and 3) (4). Different TCR Vα gene segments have preferences for binding to, and therefore being preferentially selected by, either MHC-I or MHC-II. This selection bias is dictated by their CDR1 and CDR2 sequences (5–7) and can affect the CD4^+^:CD8^+^ ratio (8). Structural analysis of TCR-pMHC complexes indicates conserved sites in TCR CDR1 and 2 of Vα and Vβ that correspond to sites on the MHC α-helices (9), which can explain these biases of selection.

After thymic selection, T cells migrate to peripheral lymphoid organs. T cell survival in the periphery requires tonic TCR signaling from self-pMHC (10, 11). This tonic signaling is also important in shaping T cell potential to mount immune responses to foreign antigens (12) and for homeostatic proliferation (1). T cells undergo lymphopenia-induced proliferation (LIP) upon transfer into lymphopenic hosts, and this process requires interactions with self-pMHC as well as signals from cytokines such as IL-7 and IL-15 (13). The probability of a particular T cell to undergo LIP correlates with its affinity for self-pMHC (10). CD5 is a negative regulator of TCR signaling, and its expression reports the strength of interaction of TCR with self-pMHC (14, 15). CD5 expression also regulates responsiveness to IL-7 in naïve T cells (16). IL-7 and IL-15 are required for homeostatic maintenance of T cells in the periphery (17, 18) and IL-7 has a role in maintenance of the TCR repertoire diversity (19).

Memory cells, marked by CD44^hi^ expression, are formed once an infection is cleared, and provide critical protection against re-encounter with the same pathogen. Apart from antigen induced memory cells, there are also “virtual” or “innate” memory phenotype (MP) cells (20, 21), which can also be identified by CD44^hi^ expression (22) and which require IL-15 for maintenance (23, 24). These cells were thought to develop mostly due to LIP in a foreign antigen-independent manner (25, 26). However, recent analysis of TCR repertoire of CD8^+^ MP cells demonstrated that TCRs expressed by MP cells are distinct from these expressed by naïve CD8^+^ T cells, with MP clones showing higher reactivity to self pMHC (27, 28), suggesting a unique development program of at least part of the CD8^+^ MP population, controlled by self-pMHC. Not all cells that undergo proliferation in response to an infection or tumor are antigen specific. Non-antigen specific bystander T cells become activated by the cytokines produced during an infection (29), such as IL-15 induced by type I interferons (30). Bystander cells display effector functions (31, 32), and play a role in protection against chronic infections in humans (33–35).

The T cell lineage-restricted protein Themis regulates the threshold between positive and negative selection of T cells in the thymus (36–40). Themis interacts with and regulates the phosphatases Shp1 (*Ptpn6*) and Shp2 (*Ptpn11*), although the precise mechanism is controversial (39, 41–43). Themis deficiency affects the metabolic response of T cells to TCR stimulation (44). Post-selection deletion of Themis reduces the homeostatic response of peripheral CD8^+^ T cells to self-pMHC (45). Themis deficiency is predicted to alter TCR repertoire but altered TCR repertoire in Themis KO mice and its functional consequences have not yet been described. Here were report comparison of TCR repertoires of Themis KO and WT mice, and the discovery of an unusual virtual memory and bystander-competent CD8^+^ subpopulation bearing Vα3.2 TCR.

## Materials and Methods

### Mice

*Themis* ^–/–^ *Foxp3-GFP*, derived from B6.129S-*Themis^tm1Gasc^* (36) crossed to B6.Cg-Foxp3^tm2Tch^/J (46), *Themis^+/+^Foxp3-GFP* (B6.Cg-Foxp3^tm2Tch^/J), *Themis^f/f^.CD4-Cre* ^–^ and *Themis^f/f^.CD4-Cre^+,^ Themis^f/f^.dLck-Cre* ^–^ and *Themis^f/f^.dLck-Cre^+^* (45), *Rag1* ^–/–^ and CD45.1 mice on C57BL/6 background were bred in our restricted flora (RF) facilities at Comparative Medicine, NUS. Mice were treated under Institutional Animal Care and Use Committee-approved guidelines in accordance with approved protocols.

### Flow Cytometry

Mice were euthanized and dissected. Peripheral (p)LN (pooled cervical, axillary, brachial, and inguinal LN) were excised and mashed upon a 70 μm cell strainer into 5 ml cRPMI. For cell surface staining, cells were spun at 1200 rpm at 4°C for 5 minutes and the resulting cell pellets were resuspended in 100 μl PBS with 0.5% BSA (FWB: FACS wash buffer), containing the fluorophore-conjugated antibodies’ dilutions (1:300) for the cell surface antigens and incubated on ice for 30 minutes in the dark. Cells were then centrifuged at 1200 rpm at 4°C for 5 minutes and resuspended in 300 μl of FWB for flow cytometry analysis. For biotinylated antibodies, staining with the antibody was followed by staining with 100 μl FWB containing BUV 395 labeled-streptavidin (BD Biosciences, New Jersey, USA) at 1:500 dilution for 30 minutes on ice. Cells were then centrifuged at 1200 rpm at 4°C for 5 minutes and resuspended in 300 μl of FWB for flow cytometry analysis.

For intracellular staining, the cells from the previous step were resuspended in 0.2 ml IC fixation buffer (eBiosciences, California, USA) while being vortexed, followed by an incubation at room temperature for 20 minutes. The cells were then washed twice with 2 ml 1X permeabilization buffer (eBiosciences, California, USA), resuspended in 100 μl FWB containing the fluorophore-conjugated antibodies’ dilutions (1:250) for the intracellular antigens and incubated at room temperature for 30 minutes. The cells were then washed once with 2 ml 1X permeabilization buffer and then with 2 ml FWB. The cells were then resuspended in 300 μl FWB for analysis on a flow cytometer. 25 μl Count Bright beads (Life Technologies, California, USA) were added to each sample for cell count analysis. Cells were analyzed on BD LSR Fortessa X-20 flow cytometer (BD Biosciences, New Jersey, USA). Flow cytometry data were analyzed using FlowJo software (Treestar, California, USA). All antibodies used for flow cytometry purposes are described in **Table 1**.

**Table 1:** Antibodies used for flow cytometry.

Antigen	Host	Target	Fluorophore	Clone	Company	Catalog no
CD4	Rat	Mouse	V450	RM4-5	eBiosciences	48-0042-82
CD4	Rat	Mouse	APC	GK1.5	eBiosciences	17-0041-83
CD5	Rat	Mouse	BV421	53-7.3	BD Biosciences	562739
CD6	Rat	Mouse	PE	M-T605	BD Biosciences	555358
CD8	Rat	Mouse	BUV395	53-6.7	BD Biosciences	563786
CD8	Rat	Mouse	APC	53-6.7	eBiosciences	17-0081-83
CD44	Rat	Mouse	BV711	IM7	BD Biosciences	563971
CD45.1	Mouse	Mouse	PE	A20	BioLegend	110707
CD45.2	Mouse	Mouse	FITC	104	eBiosciences	11-0454-82
CD49d	Rat	Mouse	PE-CF594	F344	BD Biosciences	564395
CD62l	Rat	Mouse	BV510	MEL-14	BD Biosciences	563117
CD122	Rat	Mouse	BV421	TM-β1	BD Biosciences	562960
CD218	Rat	Mouse	APC	P3TUNYA	eBiosciences	17-5183-82
IFNγ	Rat	Mouse	PE	XMG1.2	eBiosciences	12-7311-82
Ki67	Rat	Mouse	BV421	16A8	BioLegend	652411
NKG2D(CD314)	Rat	Mouse	PE-Cy7	CX5	eBiosciences	25-5882-82
Vα2	Rat	Mouse	PE	B20.1	eBiosciences	12-5812-82
Vα3.2	Rat	Mouse	APC	RR3-16	eBiosciences	17-5799-82
Vα8.3	Rat	Mouse	PE	B21.14	BD Biosciences	553377
Vα11.1, 11.2(b,d)	Rat	Mouse	PE	RR8-1	BD Biosciences	553223
Vβ2	Rat	Mouse	PE	B20.6	BD Biosciences	553281
Vβ3	Armenian Hamster	Mouse	PE	KJ25	BD Biosciences	553209
Vβ5.1,5.2	Rat	Mouse	PE	MR9-4	BioLegend	139504
Vβ6	Rat	Mouse	PE	RR4-7	BD Biosciences	553194
Vβ7	Rat	Mouse	PE	TR310	BD Biosciences	553216
Vβ8.1,8.2	Mouse	Mouse	PE	MR5-2	BD Biosciences	553186
Vβ11	Rat	Mouse	PE	CTVB11	eBiosciences	12-5817-82
Vβ12	Rat	Mouse	Biotin	RR3-15	BD Biosciences	553196

### Ki67 Staining

Cell pellets were washed twice with FWB for 5 minutes at 350g. The resulting cell pellet was vortexed after discarding the supernatant. 3 ml ice-cold ethanol was added to the cell pellet drop by drop while vortexing. Cells were votexed for another 30 seconds and then incubated at -20**°**C for an hour. The cells were then washed thrice with FWB. The resulting cells were then stained with Ki67 (BioLegend, California, USA) antibody at 1:1000 dilution and other cell surface antigens for 30 minutes at room temperature. The cells were then washed with 2 ml FWB and analyzed on a flow cytometer.

### Cell Sorting

Mice were euthanized and dissected. Lymph nodes were excised and pooled together from several mice of the same genotype to prepare the samples for sorting. These were then mashed upon a 70 μm cell strainer into 5 ml cRPMI. The resulting cell suspensions were then spun down at 1200 rpm for 5 minutes at 4°C. The resulting cell pellet was surface stained by resuspending in 0.5 ml cRPMI per mouse containing fluorescently-conjugated antibodies at 1:500 dilution, followed by incubation at 4°C for 30 minutes on a shaker. They were then washed with cRPMI and then resuspended in 0.5 ml cRPMI per mouse for sorting. The cells were sorted on either Sy2000 (Sony Corporation, Tokyo, Japan) or Facsfusion (BD Biosciences, New Jersey, USA).

### TCR Sequencing and Repertoire Analysis

For the synthesis of the NGS libraries covering entire TCR repertoires, the total RNA was isolated from similar number of sorted SP CD8 thymocytes and peripheral CD8 T cells. The Vα3.2 TCR repertoires were retrieved from sorted Vα3.2+ CD8 SP thymocytes and Vα3.2+ CD8 peripheral T cells. The reverse transcriptase reaction was performed according to the previously published protocol (47) with template switching primers TAAGAGACAGCAACTACTACTGCrGrGrG (where r indicates ribonucleotide). Two rounds of amplification of the TCR’s cDNAs were performed using Q5^®^ High-Fidelity DNA Polymerase (NEB, Massachusetts, USA) according to the manufacture instruction, with primers: tcgtcggcagcgtcagatgtgtataagagacagcaactactACTGC, GTCTCGTGGGCTCGGAGATGTGTATAAGAGACAGggtacacagcaggttctgg (first round), and CAAGCAGAAGACGGCATACGAGAT[i7]GTCTCGTGGGCTCGG AATGATACGGCGACCACCGAGATCTACAC[i5]TCGTCGGCAGCGTC where i7 and i5 represent Illumina Nextera V2 indexes (Illumina, California, USA). Libraries were purified using AMPure XP (Beckman Coulter, California, USA), and molar concentration of amplicons was quantified using Qubit DNA Assay (Thermo Fisher, Massachusetts, USA) and KAPA Library Quantification Kit (Kapa Biosystems, Massachusetts, USA). Sequencing of the TCR’s amplicons was performed on MiSeq platform using MiSeq Reagent Kits v2 (Illumina, California, USA).

Extraction of the sequences corresponding to the TCRs was performed using MiXCR platform (48). Further processing of data was done using VDJTools software (49) and Weblogo3 (50).

### CTV Labeling

Sorted CD8^+^CD44^lo^ cells were labeled with Cell Trace Violet (Life Technologies, California, USA). Cells were spun down and resuspended in PBS at a concentration of 2x10^6^/ml. Cell Trace Violet was then added to the cell suspension at the concentration of 5 μM. Cells were vortexed immediately and incubated at 37**°**C for 10 minutes while vortexing every 5 minutes. After the incubation, media was added to quench the reaction at 5 times the original staining volume and further incubated for 5 minutes at 37**°**C. The cells were then spun down at 500 g for 5 minutes at 4**°**C. The cells were then used for the subsequent experiments.

### Cytokine Stimulation

After CTV labeling, live cells were counted and adjusted to the concentration of 1*10^6^/ml. 100 μl of the cell suspension was seeded into a 96 well plate. For IL-15 stimulation, 100 μL of media with recombinant mouse IL-15 (Peprotech, New Jersey, USA) at the concentration of 2 μg/ml was added to the cells. The cells were incubated at 37**°**C for 5 days and then analyzed for proliferation by the CTV dilution on a flow cytometer. For IL-7/12/18 (Peprotech, New Jersey, USA) stimulation, 100 μl media with either IL-7 or a combination of either IL-7 and IL-12 or IL-7 and IL-18 (all at 100ng/ml) was added to the cells. The cells were incubated at 37**°**C for 7 days and then analyzed for proliferation by the CTV dilution on a flow cytometer.

### Lymphopenia Induced Proliferation Assay

After CTV labeling, live cells were counted and adjusted to the concentration of 2.5*10^6^/ml. CD45.1 recipient mice were sublethally irradiated at 6 Gy. 0.2 ml cell suspension was injected retro-orbitally (intravenous i.v.) into either *Rag1* ^–/–^ or the sublethally irradiated CD45.1 mice. 5 recipient mice were injected per donor genotype and un-injected mice were used as controls. After one week, lymph nodes and spleens were excised from the CD45.1 mice or *Rag1* ^–/–^ mice after euthanizing them. The single cell suspensions from lymph nodes and spleens were stained for the congenic markers CD45.1, CD45.2 to distinguish donor and recipient cells. The proliferation of the donor cells was analyzed based upon CTV dilution as determined using a flow cytometer.

### LM-OVA Infection

*Themis* ^–/–^ and *Themis* ^+/+^ mice were infected with 10^4^ colony-forming units of LM-OVA (51) *via* retro-orbital intravenous injection. The mice were euthanized at either day 4 or day 7 to analyze the bystander cells. Tetramer staining was done to gate out the antigen-specific cells. To assess their bystander activation, splenocytes were stimulated with IL-12 + IL-18 (Peprotech, New Jersey, USA) (100 ng/ml) for 6 hours in the presence of Brefeldin A (BD Biosciences, New Jersey, USA) at 1:500 dilution and stained for IFNγ *via* intracellular staining protocol mentioned above.

### Tetramer Preparation

3.18 μl of PE labeled Streptavidin (1 mg/ml) (Life Technologies, California, USA) was added every 10 minutes for a total of 10 times to 10 μl of 2 mg/ml biotinylated H-2 K^b^-OVA monomers in the dark. The tetramer was then used at 1:50 dilution for cell surface staining.

### Statistical Analysis

Prism (GraphPad Software, California, USA) and Excel (Microsoft Corporation, Washington, USA) were used for all statistical analysis and graphical representations. Normality of data was tested using Shapiro-Wilk test. All data sets were found to pass the normality test. Data are presented as means ± s.d., and we determined significance by two-sided Student’s t test. We considered a p-value of equal to or less than 0.05 as statistically significant.

## Results

### Biased TCR Expression in *Themis* ^–/–^ Mice

**Figure 1:**
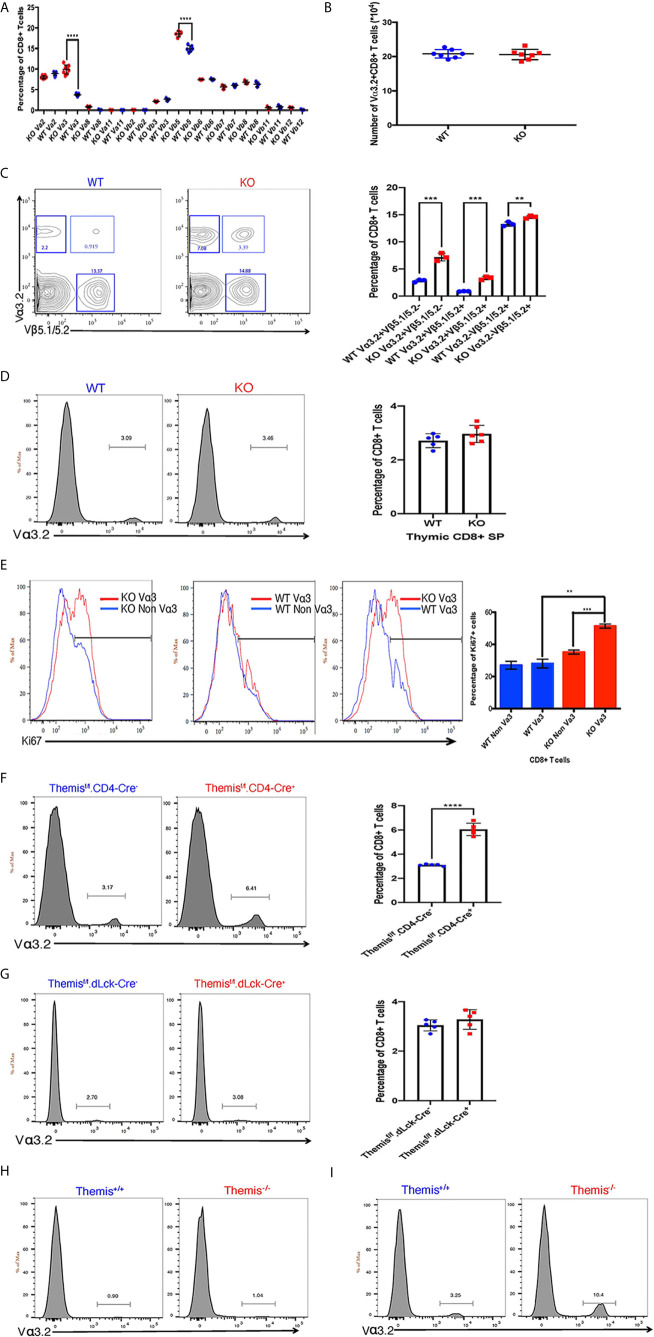
Changes in TCR repertoire of *Themis* ^–/–^ mice. **(A)** Proportion of various TCR α and β chains on CD8^+^ T cells in the periphery of *Themis* ^–/–^ (red) and *Themis* ^+/+^ (blue) mice. **(B)** Absolute numbers of Vα3.2^+^ CD8^+^ T cells in the periphery of *Themis* ^–/–^ and *Themis* ^+/+^ mice. **(C)** Proportion of Vα3.2^+^ Vβ5.1/5.2^+^ double-expressor CD8^+^ T cells in the periphery of *Themis* ^–/–^ and *Themis* ^+/+^ mice. **(D)** Proportion of Vα3.2^+^ CD8^+^ SP T cells in the thymus of *Themis* ^–/–^ and *Themis* ^+/+^ mice. **(E)** Proportion of Ki67^+^ cells amongst Vα3.2^+^ and non-Vα3.2^+^ CD8^+^ T cells in the periphery of *Themis* ^–/–^ and *Themis* ^+/+^ mice. **(F)** Proportion of Vα3.2^+^ CD8^+^ T cells in the periphery of *Themis^f/f^.CD4-Cre^-^* and *Themis^f/f^.CD4-Cre^+^* mice. **(G)** Proportion of Vα3.2^+^ CD8^+^ T cells in the periphery of *Themis^f/f^.dLck-Cre^-^* and *Themis^f/f^.dLck-Cre^+^* mice. **(H)** Proportion of Vα3.2^+^ TCR on CD4^+^ T cells in the periphery of *Themis* ^–/–^ and *Themis* ^+/+^ mice. **(I)** Proportion of Vα3.2^+^ TCR on CD8^+^ T cells in the periphery of *Themis* ^–/–^ and *Themis* ^+/+^ mice. Data representative from three independent experiments with 4-5 biological replicates per genotype per experiment. **p < 0.01, ***p < 0.001, ****p < 0.0001 as determined by two-sided Student’s t-test. All error bars represent SDs.

Because Themis regulates thymocyte selection thresholds (39, 42), we predicted that Themis germline deletion would lead to changes in TCR repertoire. T cells from peripheral lymphoid organs (lymph nodes) were analyzed for any TCR bias resulting from Themis deficiency. The total lymphocyte pool was stained with specific TCR anti-Vα and -Vβ antibodies to analyze the TCR repertoire in *Themis* ^–/–^ and *Themis* ^+/+^ mice. The biggest difference was in the proportion of Vα3.2^+^ CD8^+^ T cells (**Figure 1A**). Within the TCR Vα3 (TRAV9) family, Vα3.2 is found approximately two-fold more frequently in CD8^+^ cells than in CD4^+^ cells, whereas the other members are more frequently found in CD4^+^ cells (5, 7). The antibody RR3-16 was previously shown to recognize Vα3.2 rather than other Vα3 elements, recognizing Vα3 CDR1s with a phenylalanine at position 28 (5). This is only found in TRAV9N-3, not other TRAV9 family members [IMGT nomenclature and numbering (52)]. Thus the anti-Vα3.2 antibody RR3-16 identifies only T cells bearing a TRAV9N-3 TCR α-chain. *Themis* ^–/–^ mice had roughly three times higher proportion of Vα3.2^+^ CD8^+^ T cells compared to *Themis* ^+/+^ mice (**Figure 1A**). Although there is an increase in their percentage in *Themis* ^–/–^ mice, the total Vα3.2^+^ CD8^+^ T cell counts were the same in *Themis* ^–/–^ and *Themis* ^+/+^ mice (**Figure 1B**), despite the lymphopenia observed in *Themis* ^–/–^ mice (36–38). We also observed an increase in the proportion of Vβ5^+^ CD8^+^ T cells in *Themis* ^–/–^ mice as compared to *Themis* ^+/+^ mice (**Figure 1A**). Based on this result, we predicted an increase in the proportion of CD8^+^ T cells expressing both Vα3.2 and Vβ5.1/5.2. Indeed, the percentage of these double positive cells was approximately three times higher in *Themis* ^–/–^ mice compared to *Themis^+^* ^/+^ mice (**Figure 1C**). We did not observe differences in frequency of other TCR chains investigated, except a decrease in the proportion of Vα2^+^ CD4^+^ cells in *Themis* ^–/–^ mice relative to *Themis* ^+/+^ mice (**Supplementary Figure 1**).

To find out whether these changes in proportions of Vα3.2^+^ CD8^+^ T cells in *Themis* ^–/–^ mice originate in the thymus, we analyzed the cell surface expression of Vα3.2 in CD8 SP thymocytes. The increase in the proportion of Vα3.2^+^ CD8^+^ T cells was only observed in the periphery of *Themis* ^–/–^ mice, and not in the thymus (**Figure 1D**). We hypothesized that the increase of Vα3.2^+^ CD8^+^ T cells reflected their increased homeostatic expansion in the periphery. We analyzed expression of the proliferation marker Ki67 to estimate homeostatic proliferation of CD8^+^ T cells in the periphery. We found that a higher proportion of Vα3.2^+^ CD8^+^ T cells from *Themis* ^–/–^ mice express Ki67, compared to Vα3.2^–^ CD8^+^ T cells from *Themis* ^–/–^ mice or Vα3.2^+^ or Vα3.2^–^ CD8^+^ T cells from *Themis* ^+/+^ mice (**Figure 1E**). This suggests increased proliferation of *Themis* ^–/–^ Vα3.2^+^ CD8^+^ T cells in response to self-pMHC in the periphery.

Although the increase in proportion of Vα3.2^+^ CD8^+^ T cells was apparent only in the periphery of *Themis* ^–/–^ mice, we could not rule out that this phenotype is due to altered thymic selection in the absence of Themis. To investigate this, we used pre-selection (CD4-Cre) and post-selection (dLck-Cre) *Themis* conditional knockout mice (45). We found that only the pre-selection CD4-Cre based *Themis* deletion model showed increase in the proportion of Vα3.2^+^ CD8^+^ T cells in the periphery, relative to CD4-Cre^–^ mice (**Figure 1F**), whereas the post-selection dLck-Cre deletion model had no changes relative to dLck-Cre^–^ mice (**Figure 1G**). This shows that the phenomenon of increased proportion of Vα3.2^+^ CD8^+^ T cells has thymic origins and requires deletion of *Themis* before thymic selection.

As expected from previous studies (5–7), this TCR is more likely to be MHC-I restricted, as the prevalence of Vα3.2^+^ TCR is much higher in CD8^+^ T cells than CD4^+^ T cells in both Themis-sufficient and -deficient mice (**Figures 1H, I** **)**.

### Themis Deficiency Alters the Repertoire of Vα3.2^+^ CD8^+^ T Cells

**Figure 2:**
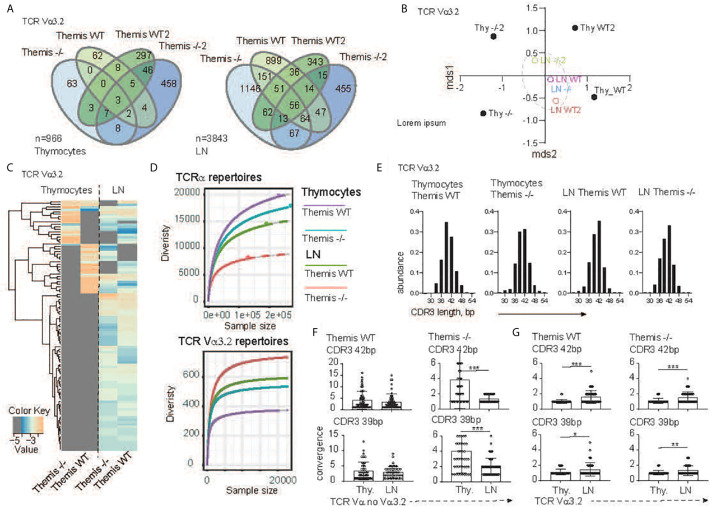
TCR sequencing analysis of the CD8 compartment from *Themis* ^+/+^ and *Themis^–/–^*mice. **(A)** The Venn diagram depicts distribution of the individual TCR Vα3.2^+^ clonotypes within SP CD8^+^ thymocytes (left) and CD8^+^ lymphocytes (right) in *Themis* ^+/+^ and *Themis^–/–^* mice. n indicates total number of detected clonotypes. **(B)** Dendrogram and non-metric multidimensional scaling (mds1 and mds2) ordination plot of *Themis* ^+/+^ and *Themis^–/–^* TCR Vα3.2^+^ repertoire similarity. **(C)** Heatmap represents abundance of the individual TCR Vα3.2^+^ clonotypes in the SP thymocytes and lymphocytes in *Themis* ^+/+^ and *Themis^–/–^* mice. **(D)** The repertoire diversity within thymocytes and peripheral T cell subsets. Upper graph. Diversity was calculated in the context of the entire TCRα repertoires. Lower panel analysis was restricted to the TCR Vα3.2^+^ (TRAV9N-3) compartment. Rarefaction curves were plotted based on a multinomial model (53) and extrapolated to the largest sample. **(E)** *In silico* spectratyping of the CDR3 region of the TCR Vα3.2^+^ compartments. CD8^+^ T cell populations and genotype are indicated on the top of each graph. TCR convergence estimated in the 50 most dominant clones with **(F)** non Vα3.2 and **(G)** Vα3.2^+^ TCRs representing 39 or 42 bp CDR3 lengths, respectively. TCR compartment, population and genotypes are indicated on the graphs. In all figures, data for each genotype were pooled from two individual experiments. Data were considered statistically significant when *p < 0.05, **p < 0.01, ***p < 0.001 as determined by for two-sided Student’s t-test with Welch’s correction.

To more precisely define the development of the TCR Vα3.2^+^ compartment in the absence of Themis, we analyzed Vα3.2 (i.e. TRAV9N-3) repertoires from SP CD8αβ^+^ thymocytes and CD8αβ^+^ lymph node T cells that developed in the *Themis* ^–/–^ and *Themis* ^+/+^ mice. After the reconstruction of TCR sequences from raw NextGen sequence datasets, we were able to identify 437 and 599 different clonotypes originating from *Themis* ^+/+^ and *Themis* ^–/–^ SP thymocytes, respectively (**Figure 2A**). The peripheral pools of TCRs in our database comprised 1751 unique clonotypes from the *Themis* ^+/+^ and 2094 from the *Themis* ^–/–^ mice. Comparison of the SP thymocytes revealed 70 public TCRs that account for 7.2% shared repertoire between these two genotypes (**Figure 2A**, left). In peripheral T cells, the number of public clonotypes rose to 473, giving 12.3% of the common component (**Figure 2A**, right). As this estimate does not consider the abundance of the individual receptor in a given repertoire, results can be biased by a large number of singletons in the datasets. However, the calculation of the similarities between *Themis* ^+/+^ and *Themis* ^–/–^ repertoires confirmed these observed trends (**Figure 2B**). Indeed, thymocyte-derived datasets were placed at a significant mathematical distance from each other (*Themis* ^+/+^ and *Themis* ^–/–^ thymocytes). In the periphery (*Themis* ^+/+^ and *Themis* ^–/–^ LN), these differences dwindled. A plausible explanation of this observed phenomenon is that analysis of the thymic repertoire is restricted to the single wave of the SP thymocytes passing through at that time. In contrast, the datasets from the lymphocytes depict a more prolonged process, which includes clonal or homeostatic proliferation together with accumulation of clones over time.

Analysis of the individual clones’ distribution indeed revealed more similarities between the T cells’ repertoires in the lymph node environment (**Figure 2C**). Importantly, many of the unique TCRs found in the *Themis* ^+/+^ and *Themis* ^–/–^ SP thymocytes acquire a public character in the peripheral lymphatic organs (**Figure 2C**). All these data collectively indicate that Vα3.2^+^ CD8^+^ T cell accumulation is a primary mechanism orchestrating peripheral repertoire development, and exclude Themis-dependent clonal deletion during the thymocytes’ progression. Themis deficiency affects the CD8^+^ compartment in both quantitative and qualitative ways (**Figure 2D**). Decrease of the total number of SP thymocytes and peripheral T cells in the *Themis* ^–/–^ model is accompanied by a substantial drop in TCR repertoire diversity within the CD8^+^ subsets when compared to the *Themis* ^+/+^ counterpart (**Figure 2D**, upper panel). Paradoxically, these relationships were inverted when we analyzed only the TCR Vα3.2^+^ compartments. In other words, the Vα3.2 repertoire was more diverse in Themis-deficient LN CD8^+^ cells than Themis-sufficient LN CD8^+^ cells. These were both more diverse than the SP thymocytes Vα3.2 repertoire, but even there, the Themis-deficient cells had more diversity than Themis-sufficient cells (**Figure 2D**, lower panel). Peripheral expansion of individual T cells results in an overall decrease of TCR repertoire diversity in the LN (**Figure 2D**, upper panel).

As individual clonotypes proliferate, the expansion process reduces the proportion of different DNA sequences for a given amino acid sequence. We term this “TCR convergence”. Thus, to test whether the Vα3.2 clonal enrichment observed in the periphery was due to accumulation of different clones rather than to clonal expansion, we analyzed TCR convergence from thymus and lymph node-derived repertoires. Because all the repertoires were dominated by clones with 39 and 42 base pair-long CDR3s (**Figure 2E**), we restricted this estimation to the 50 most dominant clones representing a given CDR3 length. In the non-Vα3.2^+^ TCR repertoire from *Themis* ^+/+^, convergence of individual TCRs was not significantly altered between thymus and LN (**Figure 2F**, left panel). In the same fractions of the CD8^+^ repertoire from *Themis* ^–/–^ mice, LN-derived TCR clonotypes had a reduced number of different DNA sequences in comparison to clonotypes associated with thymocytes (**Figure 2F**, right panel). This is likely due to LIP in the Themis-deficient mice. When we estimated TCR convergence in the Vα3.2^+^ T cell fraction, the result was strikingly different. Regardless of mouse genotype, the number of DNA sequences coding individual CDR3s increased in the peripheral repertoire (**Figure 2G**). These data strongly support the notion that the accumulation of Vα3.2^+^ T cells accounts for the repertoire enrichment in the periphery. Hence, we hypothesize that this phenomenon might be associated with better survival of the CD8^+^ Vα3.2^+^ clones, perhaps because of unique features of this TCR.

### Vα3.2^+^ CD8^+^ T Cells Have Higher Expression of CD5, CD6, and CD44 Than Non-Vα3.2^+^ CD8^+^ T Cells

**Figure 3:**
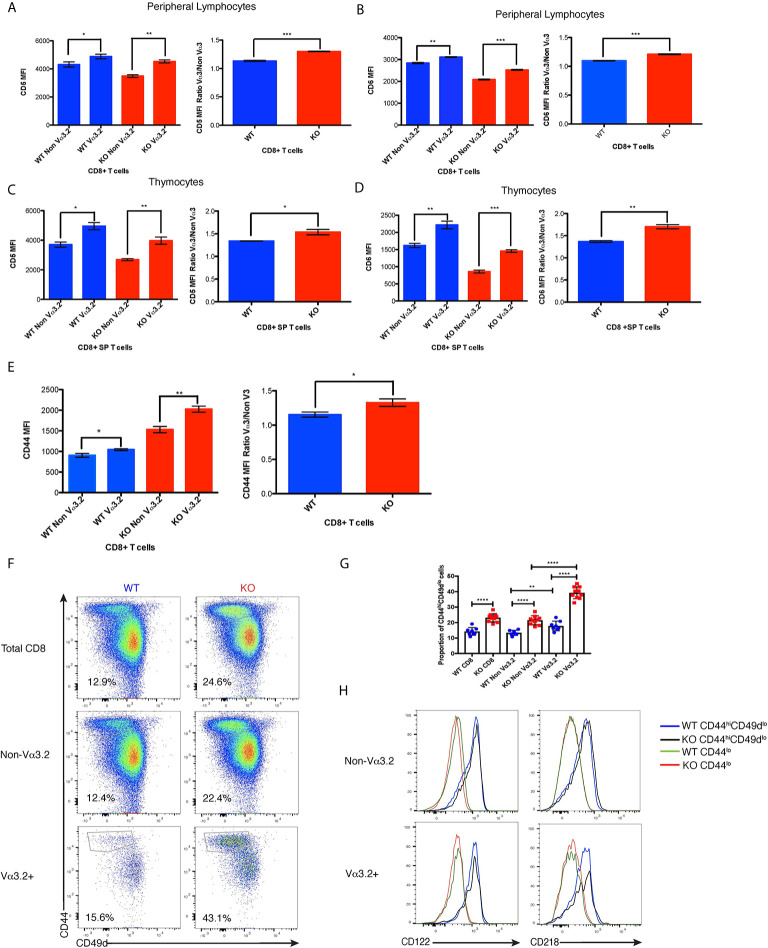
Phenotypic profile of Vα3.2^+^ CD8^+^ T cells in *Themis* ^–/–^ and *Themis* ^+/+^ mice. **(A)** CD5 expression on Vα3.2**^+^** and non-Vα3.2**^+^** CD8**^+^** T cells in the periphery of *Themis* ^–/–^ and *Themis* ^+/+^ mice. **(B)** CD6 expression on Vα3.2**^+^** and non-Vα3.2**^+^** CD8**^+^** T cells in the periphery of *Themis* ^–/–^ and *Themis* ^+/+^ mice. **(C)** CD5 expression on Vα3.2**^+^** and non-Vα3.2**^+^** CD8**^+^** SP thymocytes from *Themis* ^–/–^ and *Themis* ^+/+^ mice. **(D)** CD6 expression on Vα3.2**^+^** and non-Vα3.2**^+^** CD8**^+^** SP thymocytes from *Themis* ^–/–^ and *Themis* ^+/+^ mice. **(E)** CD44 expression on Vα3.2**^+^** and non-Vα3.2**^+^** CD8**^+^** T cells in the periphery of *Themis* ^–/–^ and *Themis* ^+/+^ mice. **(F)** Representative FACS plots for CD44 vs CD49d staining in CD8**^+^** T cells in the periphery of *Themis* ^–/–^ and *Themis* ^+/+^ mice. **(G)** Bar plots showing summary of proportion of CD44^hi^CD49d^lo^ cells in CD8**^+^** T cells in the periphery of *Themis* ^–/–^ and *Themis* ^+/+^ mice. **(H)** Histogram of CD122 and CD218 staining in CD44^hi^CD49d^lo^ vs CD44^lo^ populations of Vα3.2**^+^** and non-Vα3.2**^+^** CD8**^+^** T cells in the periphery of *Themis* ^–/–^ and *Themis* ^+/+^ mice. Data are representative of three independent experiments with 4-5 biological replicates per genotype per experiment. *p < 0.05, **p < 0.01, ***p < 0.001, ****p < 0.0001 as determined by two-sided Student’s t-test. All error bars represent SDs.

After finding that Themis deficiency induces an increase in the proportion of Vα3.2^+^ CD8^+^ T cells, we phenotyped these cells to try to understand their unique developmental and functional characteristics. Since the data from Ki67 staining indicated stronger proliferative responses to self-pMHC, we analyzed these cells for CD5 expression, a negative regulator of signaling that reports the strength of the interaction of TCR with self-pMHC (12, 14, 15). Another negative regulator of TCR signaling is CD6, a protein related to CD5. Although CD6 has not been shown to be directly involved in maintenance of homeostasis, CD6^–/–^ mice show a very similar phenotype to CD5^–/–^ mice, suggesting a similar function (54, 55). We therefore tested if CD6 can report signal from self-pMHC. Our data show that CD6 expression is correlated with that of CD5 within each of the thymocyte and lymphocyte populations analyzed, indicating that expression of CD6 and CD5 are regulated by a similar mechanism (**Supplementary Figure 2A**). Moreover, we found that reduction in signal from self pMHC through transfer of MHC class I-restricted OT-I CD8^+^ T cells into β2m deficient recipients resulted in decreased CD6 expression (**Supplementary Figure 2B**) in addition to the previously reported decrease in CD5 expression (56). These data strongly indicate that CD6 surface expression directly reports signal strength from self pMHC. CD44 is a memory and activation phenotypic marker (57), but has also been shown to correlate with CD5 expression (58–60). Thus, lymphocytes from *Themis* ^+/+^ and *Themis* ^–/–^ mice were stained with CD5, CD6 and CD44-specific antibodies and analyzed by flow cytometry. Since *Themis* ^–/–^ mice have a higher proportion of CD44^hi^ cells due to lymphopenia (36–38), we gated on CD44^lo^ cells for our analysis of CD5, CD6 and CD44 expression (**Supplementary Figure 2C**). We observed that Vα3.2^+^ cells express more CD5 and CD6 than non Vα3.2-expressing cells in both *Themis* ^+/+^ and *Themis* ^–/–^ mice, but the ratio of CD5 and CD6 expression between Vα3.2^+^ cells and non-Vα3.2 cells was even higher in *Themis* ^–/–^ mice (**Figures 3A, B** **)**. We observed even more contrasting differences in expression of CD5 and CD6 between Vα3.2^+^ and non Vα3.2-expressing CD8 SP thymocytes (**Figures 3C, D** **)**. These results suggest that Vα3.2+ cells receive stronger signal from self pMHC during development in the thymus, corroborating the findings from the conditional *Themis* deletion models, where the increased proportion of Vα3.2-expressing cells occurred if knockout happened before selection (CD4-Cre), but not if it occurred after selection (dLck-Cre) (**Figures 1F, H** **)**. These cells also express more CD44 in the periphery (**Figure 3E**), indicating a virtual memory phenotype.

As virtual memory phenotype cells have been classically defined as CD44^hi^CD49d^lo^ (24), we wanted to check whether this population would be over-represented in the Vα3.2^+^ cells. We observed that Vα3.2^+^ CD8^+^ T cells had a higher proportion of CD44^hi^CD49d^lo^ cells than non Vα3.2-expressing cells in both *Themis* ^+/+^ and *Themis* ^–/–^ mice (**Figures 3F, G** **)**. This difference was much more enhanced in *Themis* ^–/–^ mice, where almost 40% of the Vα3.2^+^ cells were CD44^hi^CD49d^lo^ as compared to only 20% of the non Vα3.2-expressing cells (**Figures 3F, G** **)**. Since virtual memory cells have been shown to be dependent on cytokine signaling for survival (24), we looked at the expression of CD122 (common gamma chain receptor for IL-2 and IL-15) and CD218 (IL-18 receptor). We observed that only the CD44^hi^CD49d^lo^ population expressed these markers (**Figure 3H**). Since a higher proportion of Vα3.2^+^ CD8^+^ T cells were CD44^hi^CD49d^lo^, and the CD44^hi^CD49d^lo^ cells express CD122 and CD218, this confirms the preferentially virtual memory phenotype of the Vα3.2^+^ CD8^+^ T cells.

### CDR3 of Vα3.2 TCRs Exhibit Unique Physical Features

The unique behavior of the TCR Vα3.2 (TRAV9N-3) repertoire and the phenotype of the Vα3.2-expressing cells raised the possibility of TCR-driven mechanisms orchestrating the function and peripheral molding of this CD8^+^ T cell compartment. The physical features of Vα segments, in particular CDR1 and CDR2, determine interactions with the α-helices of the MHC-I (5, 7, 9). At the same time, the increased amount of surface CD5 and CD6 in TCR Vα3.2^+^ CD8^+^ cells suggests enhanced interaction of these cells with self-pMHC-I. Hence, CDR3 might be involved in increased self-ligand recognition by the Vα3.2-carrying T cells (61, 62).

**Figure 4:**
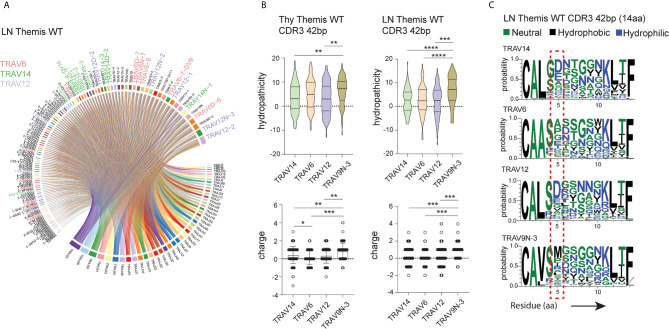
Unique physical features of CDR3 of Vα3.2^+^ CD8^+^ T cells. **(A)** Vα and Jα segment usage in the peripheral CD8^+^ TCRα repertoire. TRAV12, TRAV6, and TRAV14 segments are highlighted: lavender, plum, and green, respectively. **(B)** Hydrophobicity (upper panel) and charge (lower panel) of the CDR3 regions from Vα3.2 (TRAV9N-3) and a representative fraction of non-Vα3.2 receptors. Each Vα-associated group is represented by the 100 most dominant clonotypes from 14 amino acid-long CDR3 compartment. **(C)** Distribution of the amino acids within CDR3 regions. *p < 0.05, **p < 0.01, ***p < 0.001, ****p < 0.0001 as determined by two-sided Student’s t-test with Welch’s correction.

To shed more light on this issue, we looked more closely into the peptide sequences representing CDR3 regions. Besides TRAV9N-3 (Vα3.2), we selected TCRs that use TRAV14 (Vα2), TRAV6 (Vα4), and TRAV12 (Vα8) as a representative fraction of the lymph node- and thymus-derived CD8^+^ TCRα repertoire (**Figure 4A**, **Supplementary Figure 3A**). We restricted datasets to those TCRs with 14 amino acid-long CDR3 as these were most abundant across the investigated repertoires (**Figure 2E**). Interestingly, CDR3 sequences in Vα3.2 (TRAV9N-3) TCRs showed a distinctive physical makeup (**Figure 4B**). Estimated overall hydropathicity revealed that the CDR3s of the Vα3.2 TCRs were much more hydrophobic than those found in the other investigated repertoires. These trends were observed in both thymus and LN-derived sequences, but the differences were much stronger in the peripheral repertoires (**Figure 4B** upper panel). Similarly, CDR3s associated with Vα3.2 were more positively charged than their counterparts from non-Vα3.2 TCRs (**Figure 4B** lower panel).

To determine the source of these unique characteristics, we analyzed the distribution of amino acids within the CDR3s. In the Vα3.2^+^ TCRs, the fifth position was more frequently occupied by hydrophobic amino acids (**Figure 4C**). Methionine in this position was restricted to Vα3.2 TCRs, accounting for around 30% of the entire pool of clonotypes within the TRAV9N-3 repertoire. Isoleucine and leucine in the fifth position were not specific to Vα3.2 CDR3s, but they were much more abundant in the Vα3.2 CDR3 pool, together representing another 30% of the TRAV9N-3 repertoire. In contrast, negatively charged residues glutamic acid and, much more abundantly, aspartic acid, were frequent in the fifth position in the non-Vα3.2 TCRs (**Supplementary Figure 3B**). Finally, to test whether these observations were restricted to the investigated fraction of the repertoires, we analyzed the overall hydropathicity and charge of CDR3s (datasets were not restricted to the particular CDR3 length) from Vα3.2 and the entire non-Vα3.2^+^ TCR pool. Again, CDR3 from Vα3.2 showed a more hydrophobic and more positively charged repertoire (**Supplementary Figure 3C**).

### *Themis* ^–/–^ Vα3.2^+^ Cells Respond Better to Pro-Inflammatory Cytokine Stimulation

**Figure 5:**
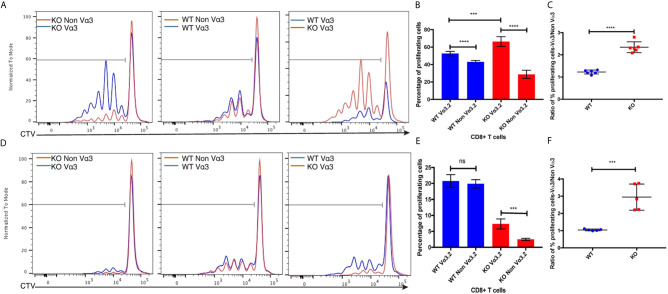
Responses of Vα3.2^+^ CD8^+^ T cells to pro-inflammatory cytokine stimulation *in vitro*. **(A)** Proliferation of Vα3.2**^+^** and non-Vα3.2**^+^** naïve CD8 T cells from *Themis* ^–/–^ and *Themis* ^+/+^ mice in response to IL-7 + IL-18 stimulation. **(B)** Histogram summary of the proliferative responses to IL-17 + IL-18 stimulation. **(C)** Ratio of % proliferating Vα3.2**^+^** and non-Vα3.2**^+^** CD8**^+^** T cells upon IL-7 + IL-18 stimulation. **(D)** Proliferation of Vα3.2**^+^** and non-Vα3.2**^+^** naïve CD8**^+^** T cells from *Themis* ^–/–^ and *Themis* ^+/+^ mice in response to IL-7 + IL-12 stimulation. **(E)** Histogram summary of the proliferative responses to IL-7 + IL-12 stimulation. **(F)** Ratio of % proliferating Vα3.2**^+^** and non-Vα3.2**^+^** CD8+ T cells upon IL-7 + IL-12 stimulation. Data representative from three independent experiments with 4-5 biological replicates per genotype per experiment. ^ns^not significant, ***p < 0.001, ****p < 0.0001 as determined by two-sided Student’s t-test. All error bars represent SDs.

Increased CD5, CD6, and CD44 surface expression suggested self-reactivity and better survival of Vα3.2^+^ CD8^+^ T cells in the periphery. Previous studies have reported that CD5^hi^ cells are hyperresponsive to *in vitro* stimulation with IL-7, and that proliferative responses to IL-7 by CD5^hi^ cells were increased upon addition of pro-inflammatory cytokines such as IL-12 and IL-18 (16, 63, 64). Therefore, we hypothesized that Vα3.2^+^ CD8^+^ T cells would be more responsive to cytokine signaling than non-Vα3.2^+^ CD8^+^ T cells, specially for Themis-deficient CD8^+^ T cells. To test this hypothesis, we sorted naïve CD8^+^ T cells from *Themis* ^–/–^ and *Themis* ^+/+^ mice and stimulated them *in vitro* in an antigen-independent manner with IL-7+IL-12, or IL-7+IL-18. Vα3.2^+^ CD8^+^ T cells proliferated better than non-Vα3.2^+^ CD8^+^ T cells, in response to IL-7+IL-18 stimulation. This difference between the two populations was stronger for Themis-deficient CD8^+^ T cells (**Figures 5A–C**). In response to IL-7+IL-12 stimulation, *Themis* ^–/–^ CD8^+^ T cells showed reduced proliferation in comparison to *Themis* ^+/+^ CD8^+^ T cells. However, we observed significantly higher proliferation of *Themis* ^–/–^ but not *Themis* ^+/+^ Vα3.2^+^ cells compared to non-Vα3.2^+^ cells (**Figures 5D–F**). These results indicate that *Themis* ^–/–^ Vα3.2^+^ CD8^+^ T cells respond more strongly to cytokines as compared to *Themis* ^–/–^ Vα3.2^-^ CD8^+^ T cells, resulting in enhanced homeostatic proliferation.

### *Themis* ^–/–^ Vα3.2^+^ CD8^+^ T Cells Undergo Enhanced LIP Compared to *Themis* ^–/–^ Non-Vα3.2^+^ CD8^+^ T Cells

**Figure 6:**
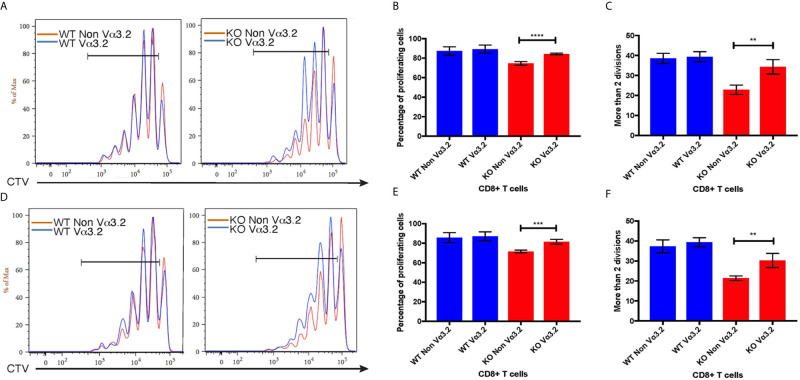
Proliferative responses of Vα3.2^+^ CD8^+^ T cells in lymphopenic hosts. Proliferation of Vα3.2**^+^** and non-Vα3.2**^+^** naïve CD8**^+^** T cells from *Themis* ^–/–^ and *Themis* ^+/+^ mice in **(A)** lymph nodes and **(D)** spleen of sublethally irradiated CD45.1hosts. Histogram summary of the proliferation responses in **(B)** lymph nodes and **(E)** spleen of sublethally irradiated CD45.1 hosts. Proportion of Vα3.2**^+^** and non-Vα3.2**^+^** CD8**^+^** T cells from *Themis* ^–/–^ and *Themis* ^+/+^ mice that had more than two divisions in **(C)** lymph nodes and **(F)** spleen of sublethally irradiated CD45.1 hosts. Data are representative from three independent experiments with 4-5 biological replicates per genotype per experiment. ^ns^not significant, **p < 0.01, ***p < 0.001, ****p < 0.0001 as determined by two-sided Student’s t-test. All error bars represent SDs.

To investigate the peripheral increase of Vα3.2^+^ CD8^+^ T cells and their LIP potential, we injected sorted naïve CD8^+^ (CD44^lo^) T cells from *Themis* ^–/–^ and *Themis* ^+/+^ mice (CD45.2) into sublethally irradiated CD45.1 recipients. After a week, the recipients were euthanized and lymph nodes and spleens were analyzed. Overall, *Themis* ^+/+^ CD8^+^ T cells showed enhanced LIP compared to *Themis* ^–/–^ CD8^+^ T cells (**Figure 6**), but in *Themis* ^–/–^ mice, the Vα3.2^+^ cells were slightly more proliferative than the non-Vα3.2^+^ cells. These results suggest better survival of Vα3.2^+^ cells in a lymphopenic environment, and explain the increase in the proportion of Vα3.2^+^ cells observed in the periphery of *Themis* ^–/–^ mice. We did not see any differences in LIP between Vα3.2^+^ and non-Vα3.2^+^ CD8^+^ T cells injected into *Rag1* ^–/–^ hosts (**Supplementary Figure 4**), possibly because proliferation in *Rag1* ^–/–^ mice is in response to the gut microbiome rather than the lymphopenia itself (65).

### Functional Relevance of Vα3.2^+^ CD8^+^ T Cells

**Figure 7:**
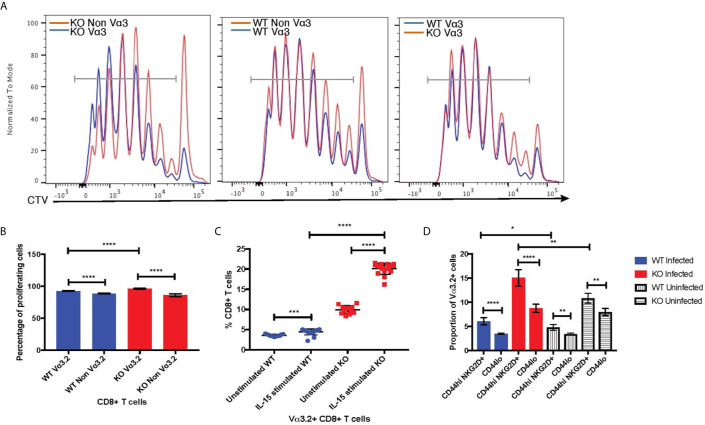
Bystander potential of Vα3.2^+^ CD8^+^ T cells. **(A)** Proliferation of Vα3.2**^+^** and Vα3.2- naïve CD8**^+^** T cells from *Themis* ^–/–^ and *Themis* ^+/+^ mice in response to IL-15 stimulation. **(B)** Histogram summary of the proliferative responses to IL-15 stimulation. **(C)** Proportion of Vα3.2**^+^** and non-Vα3.2**^+^** CD8 T cells upon IL-15 stimulation. **(D)** Proportions of Vα3.2**^+^** T cells in the bystander (CD8^+^ Tetramer^neg^ CD44^hi^ NKG2D^+^) and control (CD8^+^ Tetramer^neg^ CD44^lo^) population on day 4 of LM-OVA infection in uninfected and infected *Themis* ^–/–^ and *Themis* ^+/+^ mice. Data are representative from three independent experiments with 3-4 biological replicates per genotype per experiment. ^ns^not significant, *p < 0.05, **p < 0.01, ***p < 0.001, ****p < 0.0001 as determined by two-sided Student’s t-test. All error bars represent SDs.

The virtual memory phenotype (CD44^hi^) Vα3.2^+^ cells also suggested better responses to IL-15 stimulation, as IL-15 is required for maintenance of the memory pool (66). Thus, we tested the response of naïve CD8^+^ T cells from *Themis* ^–/–^ and *Themis* ^+/+^ mice to IL-15. In response to IL-15 stimulation *in vitro*, Vα3.2^+^ CD8^+^ T cells proliferated more than non-Vα3.2^+^ CD8^+^ T cells (**Figures 7A, B** **)**, leading to an increase in the proportion of Vα3.2^+^ cells. This increase in proportion was enhanced and much more obvious in *Themis* ^–/–^ cells compared to *Themis* ^+/+^ cells (**Figure 7C**).

Increased response to IL-15 stimulation indicates the potential of Vα3.2^+^ CD8^+^ T cells to be bystander cells. These help antigen specific cells during an infection, by becoming activated in a non-antigen specific manner by cytokines produced in the local milieu and performing effector functions to help clear the infection (67). To investigate the bystander potential in an infection model, we infected *Themis* ^–/–^ and *Themis* ^+/+^ mice with a genetically engineered strain of *Listeria monocytogenes* which expresses ovalbumin (LM-OVA) (51) and sacrificed them at day 4 to preclude antigen specific responses (68). We confirmed the absence of CD8^+^ T cells specific for OVA-derived SIINFEKL peptide at day 4 using tetramer staining; whereas SIINFEKL-specific CD8^+^ T cells were abundant on day 7 post infection (**Supplementary Figure 5A**). Bystander cells were defined as CD8^+^ Tetramer^neg^ CD44^hi^ NKG2D^+^ (**Supplementary Figures 5B, C** **)** (68). We analyzed the proportion of Vα3.2^+^ cells in this population, and compared with the proportion of Vα3.2^+^ cells in the CD8^+^ Tetramer^neg^ CD44^lo^ population as a control. We observed a higher proportion of Vα3.2^+^ cells in the bystander population compared to the control (**Figure 7D**). This effect was observed in Themis-sufficient and –deficient mice, but the magnitude was bigger in *Themis* ^–/–^ mice, possibly due to their higher proportion of Vα3.2^+^ cells and a higher proportion of CD44^hi^ cells. We identified some CD8^+^ Tetramer^neg^ CD44^hi^ NKG2D^+^ bystander phenotype cells in uninfected mice, but this population was clearly increased upon infection. To test the bystander potential of the CD8^+^ Tetramer^neg^ CD44^hi^ NKG2D^+^ cells from the infected and uninfected mice, we measured cytokine production in a non antigen-dependent manner, after IL-12+IL-18 stimulation for 6 hours (68, 69). Only bystander cells from mice infected with LM-OVA were able to produce IFN-γ, showing that the bystander effect was real and induced by infection (**Supplementary Figure 5D**). However, we did not observe any statistically significant differences between Vα3.2^+^ cells and non-Vα3.2^+^ cells in cytokine production.

## Discussion

The processes of negative and positive selection that shape the TCR repertoire occur in the thymus. Themis has been shown to be involved in these processes (36–39). Thus, we hypothesized that the TCR repertoire of *Themis* ^–/–^ mice might be different than that of *Themis* ^+/+^ mice. Our results show that there are indeed changes in TCR repertoire in *Themis* ^–/–^ mice. We observed that the proportion of Vα3.2^+^ CD8^+^ T cells and Vα3.2^+^ Vβ5.1/5.2^+^ cells was triple or more in the periphery of *Themis* ^–/–^ mice than *Themis* ^+/+^ mice. We have previously found in rearranging TCR minigene mice that co-expression of Vα3.2 and Vβ5.2 is a favored combination (70). This increase in the number of CD8^+^ T cells carrying Vα3.2 TCR occurred in the periphery, as the proportion of these cells in the thymus were similar between *Themis* ^–/–^ and *Themis* ^+/+^ mice. This peripheral increase is corroborated by their expression of Ki67, a marker of S, G2 and M phases of the cell cycle, and therefore a proliferation marker. This indicates that these CD8^+^ T cells are undergoing proliferation in the lymphopenic niche of *Themis* ^–/–^ mice, possibly in response to the same self-pMHC ligands on which they are positively selected in the thymus. As expected, we also observed that both Vα3.2^+^ and Vβ5.1/5.2^+^ TCRs were preferentially expressed in CD8^+^ T cells rather than CD4^+^ T cells. This is due to preferential selection on MHC-I rather than MHC-II after binding of CDR1 and CDR2 regions of these TCRs to MHC α-helices (5, 6). The quantitative changes in the proportion of the CD8^+^ Vα3.2^+^ and non-Vα3.2^+^ compartments in *Themis* ^–/–^ and *Themis* ^+/+^ mice were accompanied by a substantial increase in TCR repertoire diversity of the Vα3.2^+^ cells in the *Themis* ^–/–^ model. The Vα3.2 clonotype enrichment in the thymus and peripheral lymphatic organs of *Themis* ^–/–^ mice might indicate resistance of these cells to clonal deletion in the thymus and better survival in the periphery. Interestingly, observed trends in the thymic and peripheral CD8^+^ Vα3.2^+^ repertoire mirrored phenomena attributed to the central commitment and peripheral reshaping of the CD4^+^ regulatory (Treg) subset. Like the CD8^+^ Vα3.2^+^ compartment, Tregs retain a highly diverse TCR repertoire in the periphery (71, 72). Importantly, Treg selection relies on recognizing self‐antigen in the thymus (73), and self‐antigen recognition constitutes a crucial factor in further remolding of this T cell subset in the periphery (74, 75).

We analyzed the expression of CD5 on Vα3.2^+^ CD8^+^ T cells, as CD5 reports the strength of interaction with self-peptide MHC (14) and response from cytokine signals (60). Although T cells from *Themis* ^–/–^ mice had lower CD5 compared to T cells from *Themis* ^+/+^ mice (76), we found that Vα3.2^+^ CD8^+^ T cells showed higher expression of CD5 than non-Vα3.2^+^ cells in both *Themis* ^–/–^ and *Themis* ^+/+^ mice, and that the ratio of CD5 MFI between Vα3.2^+^ and Vα3.2^–^ CD8^+^ T cells was higher in *Themis* ^–/–^ mice than *Themis* ^+/+^ mice, both in the thymus and the periphery. Similar results were observed for CD6 surface expression, which we verified as a marker of signal strength from self pMHC. Thus phenotypic differences between Vα3.2^+^ and Vα3.2^–^ CD8^+^ T cells begin in the thymus. We also observed higher expression of the memory marker CD44 in peripheral Vα3.2^+^ CD8^+^ T cells compared to non-Vα3.2^+^ CD8^+^ T cells. CD44 expression has been shown to increase upon cell division in lymphopenic environments (20, 21), again indicating increased LIP by Vα3.2^+^ T cells. Data from the expression of these molecules suggest that Vα3.2^+^ T cells interact more productively with self-pMHC compared to non-Vα3.2-expressing T cells, which could explain why they are better at proliferating in the periphery, as indicated by their Ki67 expression. Taken together with our CD5 and CD6 data, it suggests that Vα3.2^+^ T cells are highly self-reactive. This notion was further supported by the analysis of CDR3 peptide sequences of Vα3.2^+^ and non-Vα3.2^+^ TCRs in the context of their physical features. Distribution of the hydrophobic residues within CDR3 constitutes unique physical makeup of Vα3.2^+^ TCRs indicating enhanced self-reactivity within this CD8^+^ T cell fraction (62).

T cells with high CD5 and CD44 expression have better responses to cytokine signaling (57, 60). We observed that, in response to IL-7+12/18 stimulation *in vitro*, we observed higher proliferative responses from Vα3.2^+^ CD8^+^ T cells compared to non-Vα3.2-expressing CD8^+^ T cells from *Themis* ^–/–^ mice. This again indicates better survival and homeostatic potential of these cells. When we injected sorted T cells into sublethally irradiated host mice, we observed that Vα3.2^+^ CD8^+^ T cells from *Themis* ^–/–^ mice showed enhanced LIP compared to their non-Vα3.2-expressing counterparts, but we did not see the same effect when we injected these cells into *Rag1* ^–/–^ hosts. This is not surprising as it has been reported that irradiated hosts are able to accumulate more donor cells than *Rag1* ^–/–^ hosts, because proliferation in *Rag1* ^–/–^ hosts is driven primarily by bacteria from the microbiome, since *Rag1* ^–/–^ hosts are severely immunodeficient (65). The enhanced LIP of Vα3.2^+^ CD8^+^ T cells from *Themis* ^–/–^ mice cells shows that they are better at survival in the periphery and possibly indicates their proliferation and maintenance by integration of strong interactions with self-peptide MHC and cytokine signaling.

In response to IL-15 stimulation, the proportion of Vα3.2^+^ CD8^+^ T cells from *Themis* ^–/–^ mice doubled in number compared to non-Vα3.2-expressing CD8^+^ T cells from *Themis* ^–/–^ mice. We saw only a slight increase in the frequency of Vα3.2^+^ T cells upon IL-15 stimulation in *Themis* ^+/+^ mice. This higher response to IL-15 stimulation in the *Themis* ^–/–^ mice indicates the potential of Vα3.2^+^ T cells to be bystanders, which help the antigen specific cells during an infection. Bystander cells become activated in a non-antigen specific manner by the cytokines produced locally, and perform effector functions to help clear the infection. Bystander cells display increased lytic capabilities and are found to be recruited to the sites of infection such as the lungs during an influenza infection (67). In recent reports, bystander cells have been shown to be involved in restraining HIV reservoir (34) and implicated in the immune response to Covid-19 (35). We tested this bystander potential in an LM-OVA model, where mice were sacrificed 4 days after infection, such that no OVA-specific cells could be detected, and bystander cells were gated based on CD44 and NKG2D expression as in previous reports (68). We observed higher proportions of Vα3.2^+^ CD8^+^ T cells in the bystander population. This phenomenon was amplified in *Themis* ^–/–^ mice, and these cells were able to produce IFN-γ upon IL-12+IL-18 stimulation in an antigen independent manner, demonstrating their bystander potential.

Overall, this work shows that the TCR repertoire is generally reduced in diversity in the absence of Themis. However, there was an unpredicted effect on T cells with a certain TCR Vα region: cells expressing Vα3.2 (TRAV9N-3), were even more frequent than usual in the CD8^+^ population in Themis-deficient mice. Their TCRα CDR3 repertoire was increased in Themis-deficient CD8^+^ peripheral T cells. Moreover, they had an unusual phenotype that indicated a stronger stimulation by self pMHC, higher responsiveness to cytokines, and an effector memory and bystander phenotype. The bystander phenotype was borne out functionally in cells responding to *Listeria* infection. Bystander T cells are commonly found during viral infections (34, 35) and tumor micro-environments (77), so a better understanding of such cytokine-responsive T cell populations with unique TCRs could help to harness them for cellular therapy against infections.

## Data Availability Statement

The datasets presented in this study can be found in online repositories. The names of the repository/repositories and accession number(s) can be found below: https://www.ncbi.nlm.nih.gov/geo/, GSE162963.

## Ethics Statement

The animal study was reviewed and approved by National University of Singapore Institutional Animal Care and Use Committee (NUS IACUC).

## Author Contributions

MP, LW, and JB performed the literature review, planned the experiments, and did data interpretation. MP performed most of the experiments. LW performed the TCR sequencing and CDR3 analysis. JB assisted in the *Listeria* infection experiments and performed the CD6 validation experiments. YC and DT assisted in the mouse breeding and irradiation of mice. JY generated the biotinylated H-2 K^b^-OVA monomers. VR and JH were involved in optimization of the experimental protocols. NG supervised the study and did data interpretation. All the authors contributed to writing and editing the manuscript. All authors contributed to the article and approved the submitted version.

## Funding

This research was supported by the Singapore Ministry of Health’s National Medical Research Council under its CBRG/0097/2015 and by the Singapore Ministry of Education’s grant 2014-T2-1-136, to NG. Work performed at Scripps Research was supported by NIH grants AI073870 and DK094173 to NG.

## Conflict of Interest

The authors declare that the research was conducted in the absence of any commercial or financial relationships that could be construed as a potential conflict of interest.

## Supplementary Material

Supplementary Figure 1 Proportion of various TCR α and β chains on CD4^+^ T cells in *Themis* ^–/–^ (red) and *Themis* ^+/+^ (blue) mice. Data are representative from three independent experiments with 4-5 biological replicates per genotype per experiment. *p<0.05, **p<0.01, ***p<0.001, ****p<0.0001 as determined by two-sided Student’s t-test. All error bars represent SDs.

Supplementary Figure 2
**(A)** Correlation between CD5 and CD6 cell surface expression on peripheral naïve CD4^+^ (CD25^–^, CD44^low^) and CD8^+^ (CD44^low^) T cells. CD5 and CD6 MFI on 10% cells with highest (CD5^high^) and lowest (CD5^low^) cell surface expression. Data from 8 mice, pooled from 2 independent experiments. **(B)** Sorted naïve (CD44^low^) OT-I CD8^+^ T cells were transferred into β_2_m WT or KO recipients, followed by CD5 and CD6 surface staining of lymphocytes 24h later. Data from 1 experiments, using 3 (β_2_m KO) and 6 (WT) recipient mice. **(C)** Gating strategy for CD44^lo^ cells from *Themis* ^–/–^ and *Themis* ^+/+^ mice. Data are representative from three independent experiments with 4-5 biological replicates per genotype per experiment. *p < 0.05, **p < 0.01, ***p < 0.001, ****p < 0.0001 as determined by two-sided Student’s t-test. All error bars represent SDs.

Supplementary Figure 3
**(A)** Vα and Jα segment usage in the thymic SP CD8 TCRα repertoire. **(B)** Distribution of the amino acids within CDR3 regions. **(C)** Hydrophobicity (upper panel) and charge (lower panel) of the CDR3 regions from Vα3.2^+^ (TRAV9N-3) and non-Vα3.2 receptors. 100 most dominant clones from entire repertoires were analyzed.

Supplementary Figure 4 Proliferation of Vα3.2**^+^** and non-Vα3.2**^+^** naïve CD8**^+^** T cells from *Themis* ^–/–^ and *Themis* ^+/+^ mice in **(A)** lymph nodes and **(B)** spleen of *Rag1* ^–/–^ hosts. Histogram summary of the proliferation responses in **(C)** lymph nodes and **(D)** spleen of *Rag1* ^–/–^ hosts. Proportion of Vα3.2**^+^** and non-Vα3.2**^+^** CD8**^+^** T cells from *Themis* ^–/–^ and *Themis* ^+/+^ mice that had more than two divisions in **(E)** lymph nodes and **(F)** spleen of *Rag1* ^–/–^ hosts. Data representative from two independent experiments with 4-5 biological replicates per genotype per experiment. *p < 0.05, **p < 0.01, ***p < 0.001, ****p < 0.0001 as determined by two-sided Student’s t-test. All error bars represent SDs.

Supplementary Figure 5
**(A)** Representative histograms of CD8^+^ Tetramer^+^ cells in uninfected and infected *Themis* ^–/–^ and *Themis* ^+/+^ mice on day 4 and day 7 of LM-OVA infection. **(B)** Gating strategy for bystander cells. **(C)** Representative FACS plots of bystander cells in uninfected and infected *Themis* ^–/–^ and *Themis* ^+/+^ mice on day 4 of LM-OVA infection. **(D)** Proportions of Vα3.2**^+^** and non-Vα3.2**^+^** CD8**^+^** T cells from uninfected and LM-OVA- infected *Themis* ^–/–^ and *Themis* ^+/+^ mice which were IFN-γ^+^ upon IL-12+18 stimulation. Data are representative from three independent experiments with 3-4 biological replicates per genotype per experiment.

## References

[1] Goldrath AW Bevan MJ. Selecting and maintaining a diverse T-cell repertoire. Nature (1999) 402:6–13. 10.1038/35005508 10580495

[2] Starr TK Jameson SC Hogquist KA. Positive and Negative selection of T cells. Annu Rev Immunol (2003) 21:139–76. 10.1146/annurev.immunol.21.120601.141107 12414722

[3] Gascoigne NR Palmer E. Signaling in thymic selection. Curr Opin Immunol (2011) 23:207–12. 10.1016/j.coi.2010.12.017 PMC3073816 21242076

[4] Davis MM. Bjorkman PJ. T-cell antigen receptor genes and T-cell recognition. Nature (1988) 334:395–402. 10.1038/334395a0 3043226

[5] Sim BC Zerva L Greene MI Gascoigne NRJ. Control of MHC Restriction by TCR Valpha CDR1 and CDR2. Science (1996) 273:963–6. 10.1126/science.273.5277.963 8688082

[6] Sim BC Lo D Gascoigne NR. Preferential expression of TCR V alpha regions in CD4/CD8 subsets: class discrimination or co-receptor recognition? Immunol Today (1998) 19:276–82. 10.1016/s0167-5699(98)01257-2 9639993

[7] Sim BC Wung JL Gascoigne NR. Polymorphism within a TCRAV family influences the repertoire through class I/II restriction. J Immunol (1998) 160:1204–11. 9570535

[8] Sim BC Aftahi N Reilly C Bogen B Schwartz RH Gascoigne NR. Thymic skewing of the CD4/CD8 ratio maps with the T-cell receptor alpha-chain locus. Curr Biol (1998) 8:701–4. 10.1016/s0960-9822(98)70276-3 9637921

[9] Garcia KC Adams JJ Feng D Ely LK. The molecular basis of TCR germline bias for MHC is surprisingly simple. Nat Immunol (2009) 10:143–7. 10.1038/ni.f.219 PMC3982143 19148199

[10] Kieper WC Burghardt JT. Surh CD. A Role for TCR Affinity in Regulating Naive T Cell Homeostasis. J Immunol (2004) 172:40–4. 10.4049/jimmunol.172.1.40 14688307

[11] Sprent CDSJ. Homeostasis of Naive and Memory T Cells. Immunity (2008) 29:848–62. 10.1016/j.immuni.2008.11.002 19100699

[12] Fulton RB Hamilton SE Xing Y Best JA Goldrath AW Hogquist KA. The TCR’s sensitivity to self peptide–MHC dictates the ability of naive CD8+ T cells to respond to foreign antigens. Nat Immunol (2014) 16:107–17. 10.1038/ni.3043 PMC4270846 25419629

[13] Tan JT Dudl E LeRoy E Murray R Sprent J Weinberg KI. IL-7 is critical for homeostatic proliferation and survival of naive T cells. Proc Natl Acad Sci USA (2001) 98:8732–7. 10.1073/pnas.161126098 PMC37504 11447288

[14] Tarakhovsky A Kanner S Hombach J Ledbetter J Muller W Killeen N. A role for CD5 in TCR-mediated signal transduction and thymocyte selection. Science (1995) 269:535–7. 10.1126/science.7542801 7542801

[15] Wong P Barton GM Forbush KA Rudensky AY. Dynamic tuning of T cell reactivity by self-peptide-major histocompatibility complex ligands. J Exp Med (2001) 193:1179–87. 10.1084/jem.193.10.1179 PMC2193333 11369789

[16] Cho J-H Kim H-O Surh CD. Sprent J. T Cell Receptor-Dependent Regulation of Lipid Rafts Controls Naive CD8+ T Cell Homeostasis. Immunity (2010) 32:214–26. 10.1016/j.immuni.2009.11.014 PMC2830358 20137986

[17] Surh CD Sprent J. Homeostatic T Cell Proliferation. J Exp Med (2000) 192:F9–F14. 10.1084/jem.192.4.F9 10952731 PMC2193242

[18] Sprent J Surh CD. Normal T cell homeostasis: the conversion of naive cells into memory-phenotype cells. Nat Immunol (2011) 12:478–84. 10.1038/ni.2018 PMC3434123 21739670

[19] Palmer MJ Mahajan VS Chen J Irvine DJ Lauffenburger DA. Signaling thresholds govern heterogeneity in IL-7-receptor-mediated responses of na&iuml;ve CD8&plus; T cells. Immunol Cell Biol (2011) 89:581–94. 10.1038/icb.2011.5 PMC3342499 21339767

[20] Kieper WC Jameson SC. Homeostatic expansion and phenotypic conversion of naïve T cells in response to self peptide/MHC ligands. Proc Natl Acad Sci USA (1999) 96:13306–11. 10.1073/pnas.96.23.13306 PMC23943 10557316

[21] Cho BK Rao VP Ge Q Eisen HN Chen J. Homeostasis-stimulated proliferation drives naive T cells to differentiate directly into memory T cells. J Exp Med (2000) 192:549–56. 10.1084/jem.192.4.549 PMC2193235 10952724

[22] Goldrath AW Bevan MJ. Low-affinity ligands for the TCR drive proliferation of mature CD8+ T cells in lymphopenic hosts. Immunity (1999) 11:183–90. 10.1016/s1074-7613(00)80093-x PMC2789737 10485653

[23] Lee J-Y Hamilton SE Akue AD Hogquist KA Jameson SC. Virtual memory CD8 T cells display unique functional properties. Proc Natl Acad Sci USA (2013) 110:13498–503. 10.1073/pnas.1307572110 PMC3746847 23898211

[24] Sosinowski T White JT Cross EW Haluszczak C Marrack P Gapin L. CD8α+ dendritic cell trans presentation of IL-15 to naive CD8+ T cells produces antigen-inexperienced T cells in the periphery with memory phenotype and function. J Immunol (2013) 190:1936–47. 10.4049/jimmunol.1203149 PMC3578102 23355737

[25] Akue AD Lee J-Y Jameson SC. Derivation and maintenance of virtual memory CD8 T cells. J Immunol (2012) 188:2516–23. 10.4049/jimmunol.1102213 PMC3294185 22308307

[26] Jameson SC Lee YJ Hogquist KA. Innate memory T cells. Adv Immunol (2015) 126:173–213. 10.1016/bs.ai.2014.12.001 25727290 PMC4670568

[27] Drobek A Moudra A Mueller D Huranova M Horkova V Pribikova M. Strong homeostatic TCR signals induce formation of self-tolerant virtual memory CD8 T cells. EMBO J (2018) 37:3236. 10.15252/embj.201798518 PMC6043851 29752423

[28] Miller CH Klawon DEJ Zeng S Lee V Socci ND Savage PA. Eomes identifies thymic precursors of self-specific memory-phenotype CD8+ T cells. Nat Immunol (2020) 21:567–77. 10.1038/s41590-020-0653-1 PMC7193531 32284593

[29] Koschella M Voehringer D Pircher H. CD40 Ligation In Vivo Induces Bystander Proliferation of Memory Phenotype CD8 T Cells. J Immunol (2004) 172:4804–11. 10.4049/jimmunol.172.8.4804 15067057

[30] Tough DF Borrow P Sprent J. Induction of bystander T cell proliferation by viruses and type I interferon *in vivo* . Science (1996) 272:1947–50. 10.1126/science.272.5270.1947 8658169

[31] Monjazeb AM Tietze JK Grossenbacher SK Hsiao H-H Zamora AE Mirsoian A. Bystander Activation and Anti-Tumor Effects of CD8+ T Cells Following Interleukin-2 Based Immunotherapy Is Independent of CD4+ T Cell Help. PloS One (2014) 9:e102709–13. 10.1371/journal.pone.0102709 PMC4131875 25119341

[32] Rivino L Kumaran EA Thein T-L Too CT Gan VCH Hanson BJ. Virus-specific T lymphocytes home to the skin during natural dengue infection. Sci Transl Med (2015) 7:278ra35–278ra35. 10.1126/scitranslmed.aaa0526 25761891

[33] Chen AM Khanna N Stohlman SA Bergmann CC. Virus-specific and bystander CD8 T cells recruited during virus-induced encephalomyelitis. J Virol (2005) 79:4700–8. 10.1128/JVI.79.8.4700-4708.2005 PMC1069536 15795256

[34] Jin J-H Huang H-H Zhou M-J Li J Hu W Huang L. Virtual memory CD8+ T cells restrain the viral reservoir in HIV-1-infected patients with antiretroviral therapy through derepressing KIR-mediated inhibition. Cell Mol Immunol (2020) 376:49. 10.1038/s41423-020-0408-9 PMC7784989 32210395

[35] Mathew D Giles JR Baxter AE Oldridge DA Greenplate AR Wu JE. Deep immune profiling of COVID-19 patients reveals distinct immunotypes with therapeutic implications. Science (2020) 369:eabc8511. 10.1126/science.abc8511 32669297 PMC7402624

[36] Fu G Vallée S Rybakin V McGuire MV Ampudia J Brockmeyer C. Themis controls thymocyte selection through regulation of T cell antigen receptor–mediated signaling. Nat Immunol (2009) 10:848–56. 10.1038/ni.1766 PMC2757056 19597499

[37] Johnson AL Aravind L Shulzhenko N Morgun A Choi S-Y Crockford TL. Themis is a member of a new metazoan gene family and is required for the completion of thymocyte positive selection. Nat Immunol (2009) 10:831–9. 10.1038/ni.1769 PMC2908989 19597497

[38] Lesourne R Uehara S Lee J Song K-D Li L Pinkhasov J. Themis, a T cell-specific protein important for late thymocyte development. Nat Immunol (2009) 10:840–7. 10.1038/ni.1768 PMC2848698 19597498

[39] Fu G Casas J Rigaud S Rybakin V Lambolez F Brzostek J. Themis sets the signal threshold for positive and negative selection in T-cell development. Nature (2013) 504:441–5. 10.1038/nature12718 PMC3977001 24226767

[40] Gascoigne NRJ Rybakin V Acuto O Brzostek J. TCR Signal Strength and T Cell Development. Annu Rev Cell Dev Biol (2016) 32:327–48. 10.1146/annurev-cellbio-111315-125324 27712102

[41] Paster W Bruger AM Katsch K Grégoire C Roncagalli R Fu G. A THEMIS: SHP1 complex promotes T-cell survival. EMBO J (2015) 34:393–409. 10.15252/embj.201387725 25535246 PMC4339124

[42] Choi S Warzecha C Zvezdova E Lee J Argenty J Lesourne R. THEMIS enhances TCR signaling and enables positive selection by selective inhibition of the phosphatase SHP-1. Nat Immunol (2017) 18:433–41. 10.1038/ni.3692 PMC5807080 28250424

[43] Mehta M Brzostek J Chen EW Tung DWH Chen S Sankaran S. Themis-associated phosphatase activity controls signaling in T cell development. Proc Natl Acad Sci USA (2018) 115:E11331–40. 10.1073/pnas.1720209115 PMC6275480 30413615

[44] Prasad M Brzostek J Gautam N Balyan R Rybakin V Gascoigne NRJ. Themis regulates metabolic signaling and effector functions in CD4+ T cells by controlling NFAT nuclear translocation. Cell Mol Immunol (2020) 17:364. 10.1038/s41423-020-00578-4 PMC8429700 33177694

[45] Brzostek J Gautam N Zhao X Chen EW Mehta M Tung DWH. T cell receptor and cytokine signal integration in CD8+ T cells is mediated by the protein Themis. Nat Immunol (2020) 21:186–98. 10.1038/s41590-019-0570-3 31932808

[46] Lin W Haribhai D Relland LM Truong N Carlson MR Williams CB. Regulatory T cell development in the absence of functional Foxp3. Nat Immunol (2007) 8:359–68. 10.1038/ni1445 17273171

[47] Matz M Shagin D Bogdanova E Britanova O Lukyanov S Diatchenko L. Amplification of cDNA ends based on template-switching effect and step-out PCR. Nucleic Acids Res (1999) 27:1558–60. 10.1093/nar/27.6.1558 PMC148354 10037822

[48] Bolotin DA Poslavsky S Mitrophanov I Shugay M Mamedov IZ Putintseva EV. MiXCR: software for comprehensive adaptive immunity profiling. Nat Methods (2015) 12:380–1. 10.1038/nmeth.3364 25924071

[49] Shugay M Bagaev DV Turchaninova MA Bolotin DA Britanova OV Putintseva EV. VDJtools: Unifying Post-analysis of T Cell Receptor Repertoires. PloS Comput Biol (2015) 11:e1004503. 10.1371/journal.pcbi.1004503 26606115 PMC4659587

[50] Crooks GE Hon G Chandonia J-M Brenner SE. WebLogo: a sequence logo generator. Genome Res (2004) 14:1188–90. 10.1101/gr.849004 PMC419797 15173120

[51] Pope C Kim SK Marzo A Masopust D Williams K Jiang J. Organ-specific regulation of the CD8 T cell response to Listeria monocytogenes infection. J Immunol (2001) 166:3402–9. 10.4049/jimmunol.166.5.3402 11207297

[52] Lefranc M-P Giudicelli V Duroux P Jabado-Michaloud J Folch G Aouinti S. IMGT^®^, the international ImMunoGeneTics information system^®^ 25 years on. Nucleic Acids Res (2015) 43:D413–22. 10.1093/nar/gku1056 PMC4383898 25378316

[53] Colwell RK Chao A Gotelli NJ Lin SY Mao CX Chazdon RL. Models and estimators linking individual-based and sample-based rarefaction, extrapolation and comparison of assemblages. J Plant Ecol (2012) 5:3–21. 10.1093/jpe/rtr044

[54] Orta-Mascaró M Consuegra-Fernández M Carreras E Roncagalli R Carreras-Sureda A Alvarez P. CD6 modulates thymocyte selection and peripheral T cell homeostasis. J Exp Med (2016) 213:1387–97. 10.1084/jem.20151785 PMC4986531 27377588

[55] Gimferrer I Farnós M Calvo M Mittelbrunn M Enrich C Sánchez-Madrid F. The Accessory Molecules CD5 and CD6 Associate on the Membrane of Lymphoid T Cells. J Biol Chem (2003) 278:8564–71. 10.1074/jbc.M209591200 12473675

[56] Azzam HS Grinberg A Lui K Shen H Shores EW Love PE. CD5 Expression Is Developmentally Regulated By T Cell Receptor (TCR) Signals and TCR Avidity. J Exp Med (1998) 188:2301–11. 10.1084/jem.188.12.2301 PMC2212429 9858516

[57] Ponta H Sherman L Herrlich PA. CD44: From adhesion molecules to signalling regulators. Nat Rev Mol Cell Biol (2003) 4:33–45. 10.1038/nrm1004 12511867

[58] Mitnacht R Tacke M Hünig T. Expression of cell interaction molecules by immature rat thymocytes during passage through the CD4+8+ compartment: developmental regulation and induction by T cell receptor engagement of CD2, CD5, CD28, CD11a, CD44 and CD53. Eur J Immunol (1995) 25:328–32. 10.1002/eji.1830250204 7533082

[59] Kassiotis G Zamoyska R Stockinger B. Involvement of avidity for major histocompatibility complex in homeostasis of naive and memory T cells. J Exp Med (2003) 197:1007–16. 10.1084/jem.20021812 PMC2193871 12707300

[60] Herndler-Brandstetter D Brunner S Weiskopf D van Rijn R Landgraf K Dejaco C. Post-thymic regulation of CD5 levels in human memory T cells is inversely associated with the strength of responsiveness to interleukin-15. Hum Immunol (2011) 72:627–31. 10.1016/j.humimm.2011.03.028 PMC3144390 21539877

[61] Ritmahan W Kesmir C Vroomans RMA. Revealing factors determining immunodominant responses against dominant epitopes. Immunogenetics (2020) 72:109–18. 10.1007/s00251-019-01134-9 PMC6971151 31811313

[62] Stadinski BD Shekhar K Gómez-Touriño I Jung J Sasaki K Sewell AK. Hydrophobic CDR3 residues promote the development of self-reactive T cells. Nat Immunol (2016) 17:946–55. 10.1038/ni.3491 PMC4955740 27348411

[63] Walsh MC Pearce EL Cejas PJ Lee J Wang L-S Choi Y. IL-18 synergizes with IL-7 to drive slow proliferation of naive CD8 T cells by costimulating self-peptide-mediated TCR signals. J Immunol (2014) 193:3992–4001. 10.4049/jimmunol.1400396 25200954 PMC4185248

[64] Goplen NP Saxena V Knudson KM Schrum AG Gil D Daniels MA. IL-12 Signals through the TCR To Support CD8 Innate Immune Responses. J Immunol (2016) 197:2434–43. 10.4049/jimmunol.1600037 PMC5010952 27521342

[65] Kieper WC Troy A Burghardt JT Ramsey C Lee JY Jiang H-Q. Recent immune status determines the source of antigens that drive homeostatic T cell expansion. J Immunol (2005) 174:3158–63. 10.4049/jimmunol.174.6.3158 15749843

[66] Zhang X Sun S Hwang I Tough DF Sprent J. Potent and selective stimulation of memory-phenotype CD8+ T cells *in vivo* by IL-15. Immunity (1998) 8:591–9. 10.1016/s1074-7613(00)80564-6 9620680

[67] Sckisel GD Tietze JK Zamora AE Hsiao H-H Priest SO Wilkins DEC. Influenza infection results in local expansion of memory CD8 +T cells with antigen non-specific phenotype and function. Clin Exp Immunol (2013) 175:79–91. 10.1111/cei.12186 PMC3898557 23937663

[68] Chu T Tyznik AJ Roepke S Berkley AM Woodward-Davis A Pattacini L. Bystander-Activated Memory CD8+ T Cells Control Early Pathogen Load in an Innate-like, NKG2D-Dependent Manner. Cell Rep (2013) 3:701–8. 10.1016/j.celrep.2013.02.020 PMC3628815 23523350

[69] Freeman BE Hammarlund E Raué H-P Slifka MK. Regulation of innate CD8+ T-cell activation mediated by cytokines. Proc Natl Acad Sci USA (2012) 109:9971–6. 10.1073/pnas.1203543109 PMC3382521 22665806

[70] Rybakin V Westernberg L Fu G Kim H-O Ampudia J Sauer K. Allelic Exclusion of TCR α-Chains upon Severe Restriction of Vα Repertoire. PloS One (2014) 9:e114320–15. 10.1371/journal.pone.0114320 PMC4264757 25500569

[71] Leung MWL Shen S Lafaille JJ. TCR-dependent differentiation of thymic Foxp3+ cells is limited to small clonal sizes. J Exp Med (2009) 206:2121–30. 10.1084/jem.20091033 PMC2757883 19737865

[72] Föhse L Suffner J Suhre K Wahl B Lindner C Lee C-W. High TCR diversity ensures optimal function and homeostasis of Foxp3+ regulatory T cells. Eur J Immunol (2011) 41:3101–13. 10.1002/eji.201141986 21932448

[73] Jordan MS Boesteanu A Reed AJ Petrone AL Holenbeck AE Lerman MA. Thymic selection of CD4+CD25+ regulatory T cells induced by an agonist self-peptide. Nat Immunol (2001) 2:301–6. 10.1038/86302 11276200

[74] Romagnoli P Hudrisier D van Meerwijk JPM. Preferential recognition of self antigens despite normal thymic deletion of CD4(+)CD25(+) regulatory T cells. J Immunol (2002) 168:1644–8. 10.4049/jimmunol.168.4.1644 11823492

[75] Wong J Obst R Correia-Neves M Losyev G Mathis D Benoist C. Adaptation of TCR repertoires to self-peptides in regulatory and nonregulatory CD4+ T cells. J Immunol (2007) 178:7032–41. 10.4049/jimmunol.178.11.7032 17513752

[76] Zvezdova E Mikolajczak J Garreau A Marcellin M Rigal L Lee J. Themis1 enhances T cell receptor signaling during thymocyte development by promoting Vav1 activity and Grb2 stability. Sci Signal (2016) 9:ra51–1. 10.1126/scisignal.aad1576 27188442

[77] Simoni Y Becht E Fehlings M Loh CY Koo S-L Teng KWW. Bystander CD8+ T cells are abundant and phenotypically distinct in human tumour infiltrates. Nature (2018) 557:575–9. 10.1038/s41586-018-0130-2 29769722

