# Supplementary figures and images for: Expansion of an Unusual Virtual Memory CD8+ Subpopulation Bearing Vα3.2 TCR in Themis-Deficient Mice
Source: Front Immunol. 2021 Apr 7;12:644483. doi: 10.3389/fimmu.2021.644483 (PMC8058184; doi:10.3389/fimmu.2021.644483)

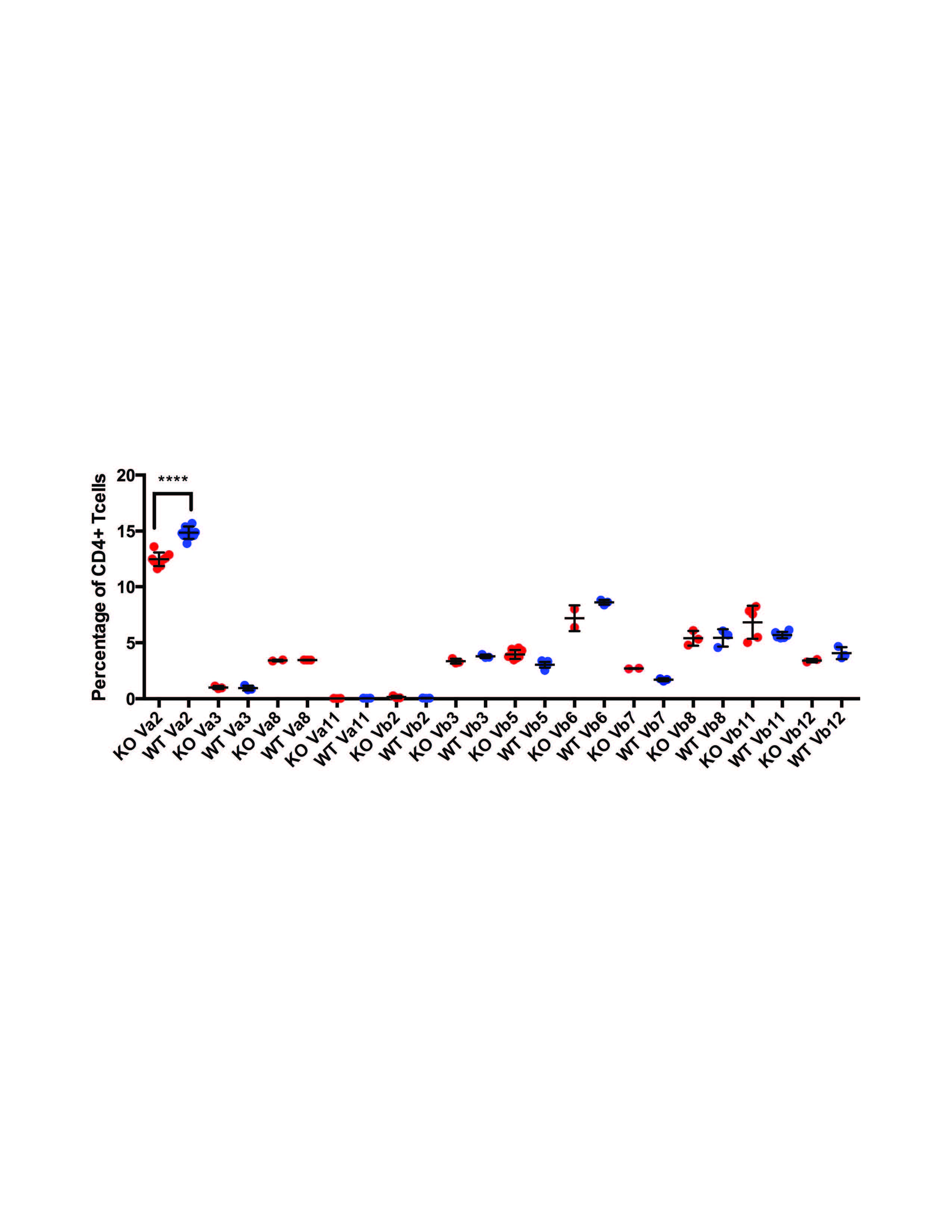

Supplement: Supplementary Figure 1 — Proportion of various TCR α and β chains on CD4+ T cells in Themis –/– (red) and Themis +/+ (blue) mice. Data are representative from three independent experiments with 4-5 biological replicates per genotype per experiment. *p<0.05, **p<0.01, ***p<0.001, ****p<0.0001 as determined by two-sided Student’s t-test. All error bars represent SDs. [file Image_1.jpeg]

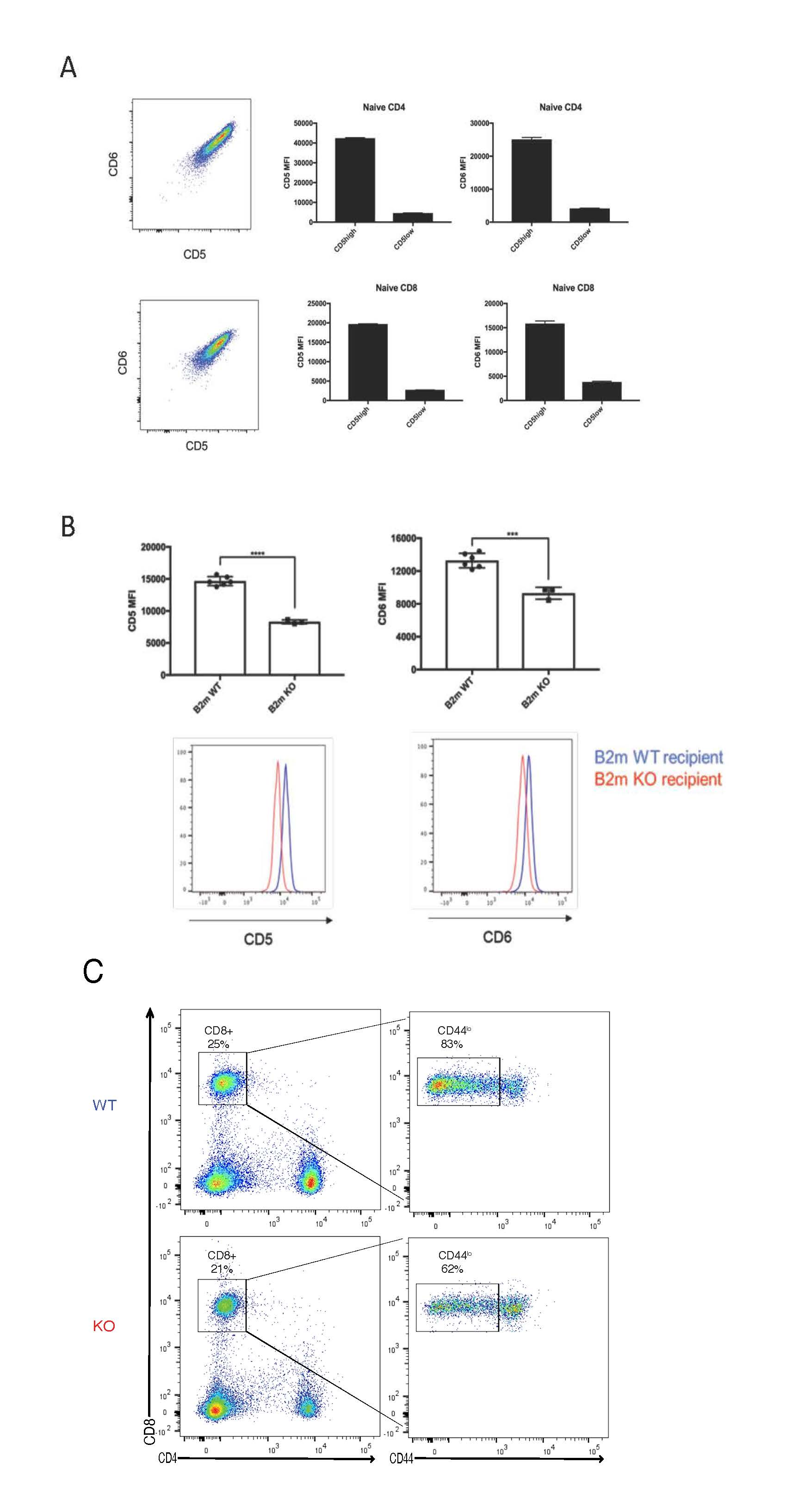

Supplement: Supplementary Figure 2 — (A) Correlation between CD5 and CD6 cell surface expression on peripheral naïve CD4+ (CD25–, CD44low) and CD8+ (CD44low) T cells. CD5 and CD6 MFI on 10% cells with highest (CD5high) and lowest (CD5low) cell surface expression. Data from 8 mice, pooled from 2 independent experiments. (B) Sorted naïve (CD44low) OT-I CD8+ T cells were transferred into β2m WT or KO recipients, followed by CD5 and CD6 surface staining of lymphocytes 24h later. Data from 1 experiments, using 3 (β2m KO) and 6 (WT) recipient mice. (C) Gating strategy for CD44lo cells from Themis –/– and Themis +/+ mice. Data are representative from three independent experiments with 4-5 biological replicates per genotype per experiment. *p < 0.05, **p < 0.01, ***p < 0.001, ****p < 0.0001 as determined by two-sided Student’s t-test. All error bars represent SDs. [file Image_2.jpeg]

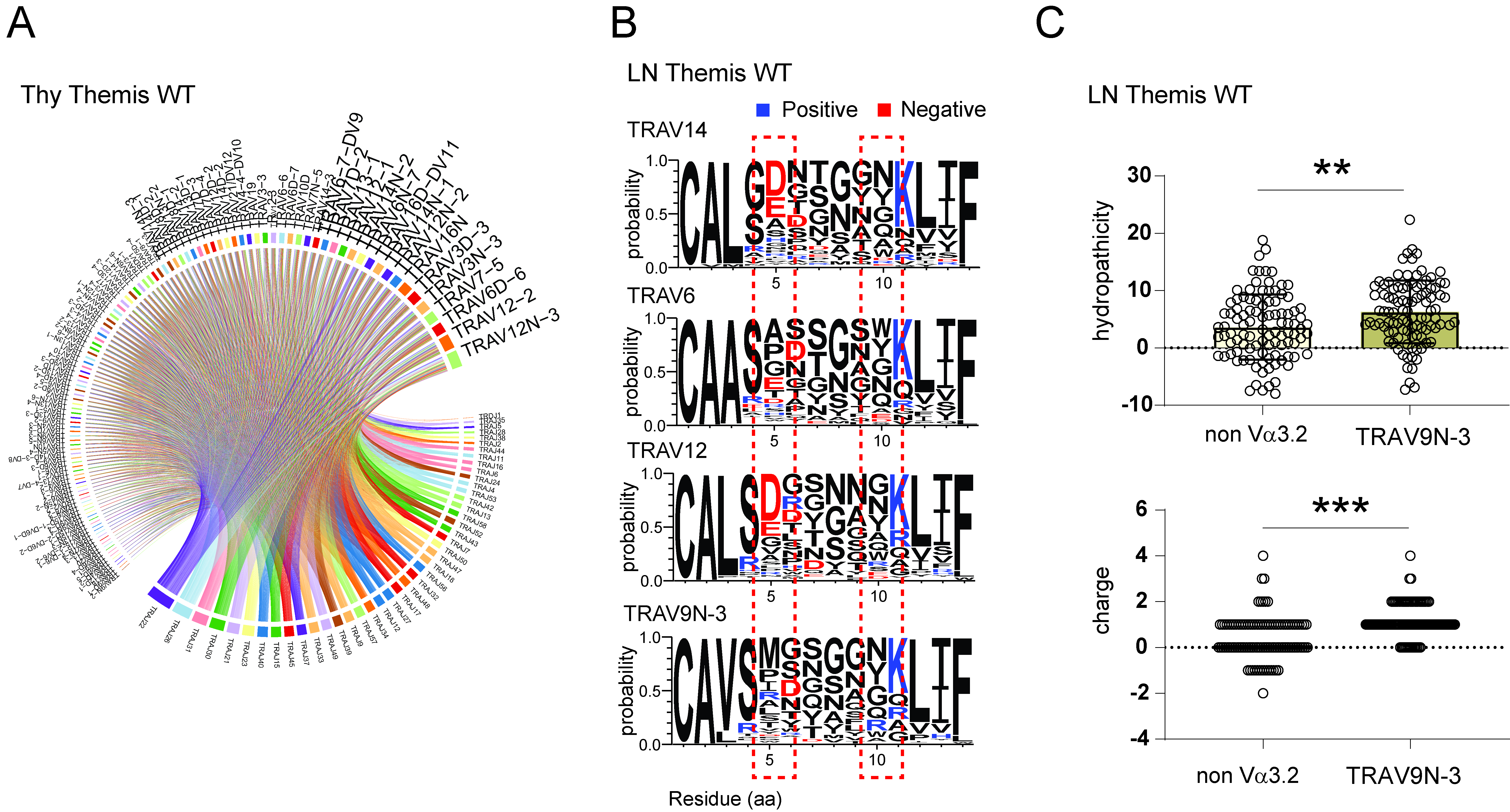

Supplement: Supplementary Figure 3 — (A) Vα and Jα segment usage in the thymic SP CD8 TCRα repertoire. (B) Distribution of the amino acids within CDR3 regions. (C) Hydrophobicity (upper panel) and charge (lower panel) of the CDR3 regions from Vα3.2+ (TRAV9N-3) and non-Vα3.2 receptors. 100 most dominant clones from entire repertoires were analyzed. [file Image_3.jpeg]

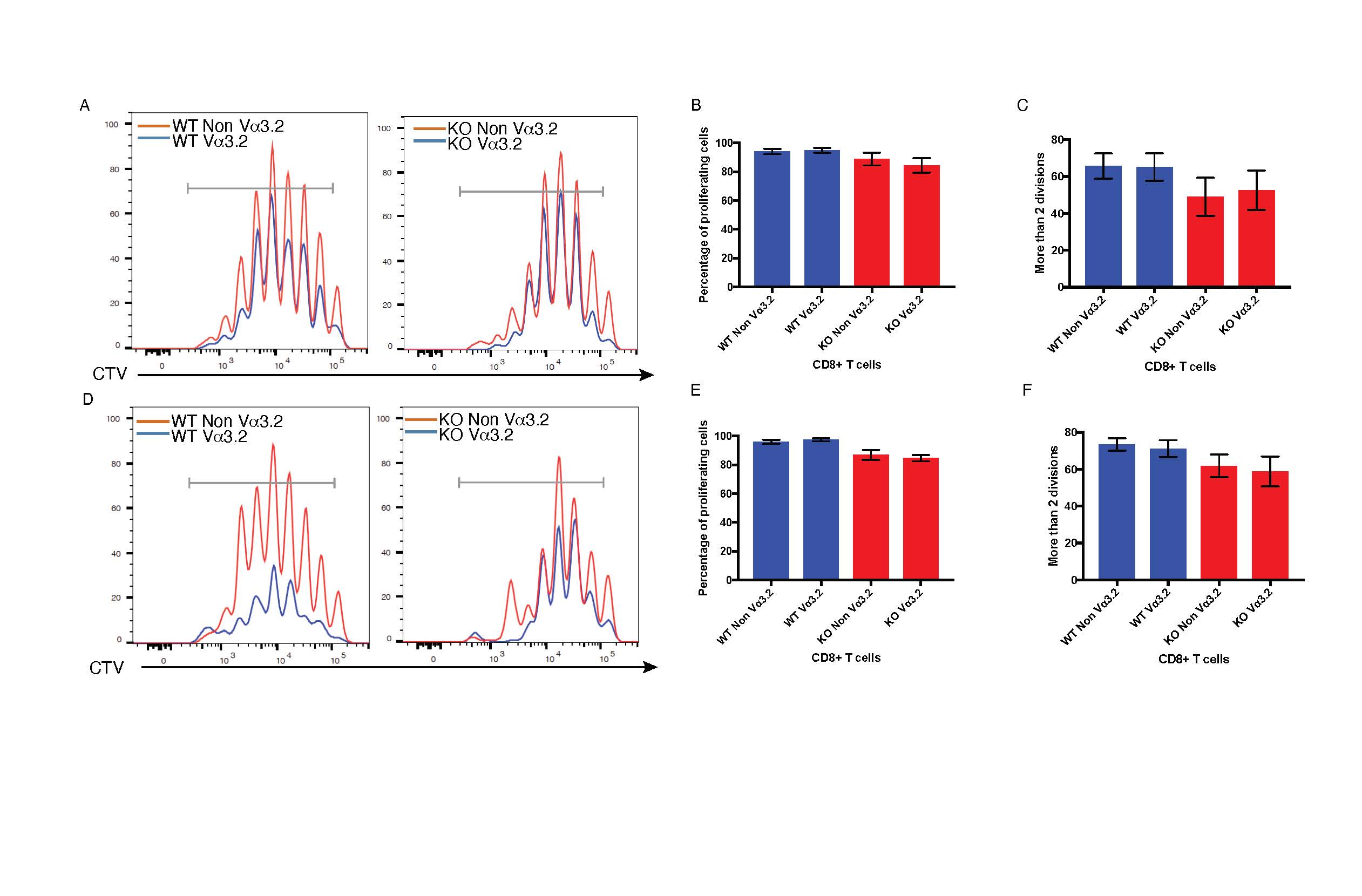

Supplement: Supplementary Figure 4 — Proliferation of Vα3.2+ and non-Vα3.2+ naïve CD8+ T cells from Themis –/– and Themis +/+ mice in (A) lymph nodes and (B) spleen of Rag1 –/– hosts. Histogram summary of the proliferation responses in (C) lymph nodes and (D) spleen of Rag1 –/– hosts. Proportion of Vα3.2+ and non-Vα3.2+ CD8+ T cells from Themis –/– and Themis +/+ mice that had more than two divisions in (E) lymph nodes and (F) spleen of Rag1 –/– hosts. Data representative from two independent experiments with 4-5 biological replicates per genotype per experiment. *p < 0.05, **p < 0.01, ***p < 0.001, ****p < 0.0001 as determined by two-sided Student’s t-test. All error bars represent SDs. [file Image_4.jpeg]

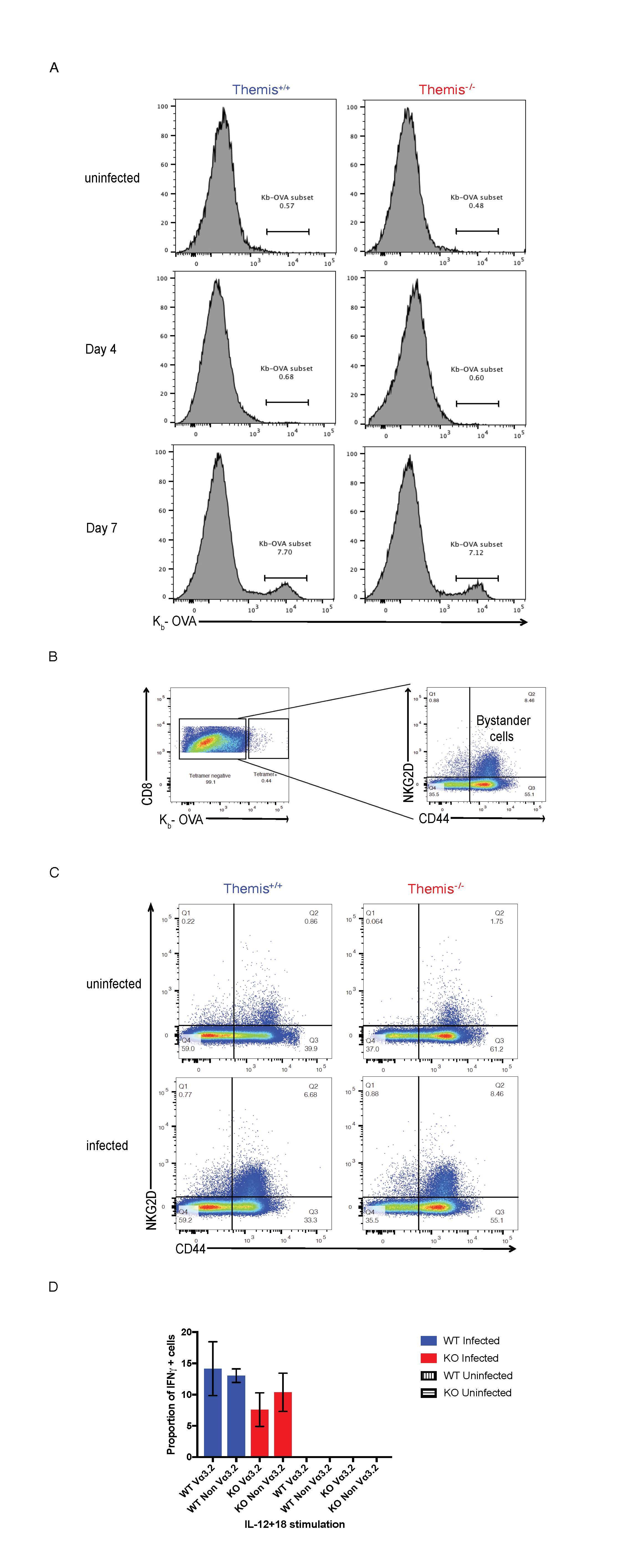

Supplement: Supplementary Figure 5 — (A) Representative histograms of CD8+ Tetramer+ cells in uninfected and infected Themis –/– and Themis +/+ mice on day 4 and day 7 of LM-OVA infection. (B) Gating strategy for bystander cells. (C) Representative FACS plots of bystander cells in uninfected and infected Themis –/– and Themis +/+ mice on day 4 of LM-OVA infection. (D) Proportions of Vα3.2+ and non-Vα3.2+ CD8+ T cells from uninfected and LM-OVA- infected Themis –/– and Themis +/+ mice which were IFN-γ+ upon IL-12+18 stimulation. Data are representative from three independent experiments with 3-4 biological replicates per genotype per experiment. [file Image_5.jpeg]
